# Natural human Bet v 1‐specific IgG antibodies recognize non‐conformational epitopes whereas IgE reacts with conformational epitopes

**DOI:** 10.1111/all.15865

**Published:** 2023-09-13

**Authors:** Georgii Brazhnikov, Evgenii Smolnikov, Alla Litovkina, Tianchi Jiang, Artem Shatilov, Inna Tulaeva, Mikhail Tulaev, Alexander Karaulov, Alina Poroshina, Yury Zhernov, Margarete Focke‐Tejkl, Milena Weber, Oluwatoyin Akinfenwa, Olga Elisyutina, Sergey Andreev, Igor Shilovskiy, Nadezhda Shershakova, Valeriy Smirnov, Elena Fedenko, Tatiana Sergeevna Lepeshkova, Evgeny Cronidovich Beltyukov, Veronika Victorovna Naumova, Michael Kundi, Musa Khaitov, Ursula Wiedermann, Rudolf Valenta, Raffaela Campana

**Affiliations:** ^1^ Division of Immunopathology, Department of Pathophysiology and Allergy Research, Center for Pathophysiology, Infectiology and Immunology Medical University of Vienna Vienna Austria; ^2^ Institute for Specific Prophylaxis and Tropical Medicine, Center for Pathophysiology, Infectiology and Immunology Medical University of Vienna Vienna Austria; ^3^ National Research Center Institute of Immunology Federal Medical‐Biological Agency of Russia Moscow Russia; ^4^ Department of Immunology, Institute of Medicine RUDN University Moscow Russia; ^5^ Laboratory of Immunopathology, Department of Clinical Immunology and Allergology I.M. Sechenov First Moscow State Medical University (Sechenov University) Moscow Russia; ^6^ F. Erismann Institute of Public Health I.M. Sechenov First Moscow State Medical University (Sechenov University) Moscow Russia; ^7^ Karl Landsteiner University of Health Sciences Krems Austria; ^8^ Department of Polyclinic Pediatrics Ural State Medical University Yekaterinburg Russia; ^9^ Department of Faculty Therapy, Endocrinology, Allergology and Immunology Ural State Medical University Yekaterinburg Russia; ^10^ Institute for Hygiene and Applied Immunology, Center for Public Health Medical University of Vienna Vienna Austria; ^11^ Pirogov Russian National Research Medical University Moscow Russia

**Keywords:** allergen, allergen‐specific IgE, allergy, Bet v 1, birch pollen allergy, epitope, IgG

## Abstract

**Background:**

The nature of epitopes on Bet v 1 recognized by natural IgG antibodies of birch pollen allergic patients and birch pollen‐exposed but non‐sensitized subjects has not been studied in detail.

**Objective:**

To investigate IgE and IgG recognition of Bet v 1 and to study the effects of natural Bet v 1‐specific IgG antibodies on IgE recognition of Bet v 1 and Bet v 1‐induced basophil activation.

**Methods:**

Sera from birch pollen allergic patients (BPA, *n* = 76), allergic patients without birch pollen allergy (NBPA, *n* = 40) and non‐allergic individuals (NA, *n* = 48) were tested for IgE, IgG as well as IgG_1_ and IgG_4_ reactivity to folded recombinant Bet v 1, two unfolded recombinant Bet v 1 fragments comprising the N‐terminal (F1) and C‐terminal half of Bet v 1 (F2) and unfolded peptides spanning the corresponding sequences of Bet v 1 and the apple allergen Mal d 1 by ELISA or micro‐array analysis. The ability of Bet v 1‐specific serum antibodies from non‐allergic subjects to inhibit allergic patients IgE or IgG binding to rBet v 1 or to unfolded Bet v 1‐derivatives was assessed by competition ELISAs. Furthermore, the ability of serum antibodies from allergic and non‐allergic subjects to modulate Bet v 1‐induced basophil activation was investigated using rat basophilic leukaemia cells expressing the human FcεRI which had been loaded with IgE from BPA patients.

**Results:**

IgE antibodies from BPA patients react almost exclusively with conformational epitopes whereas IgG, IgG_1_ and IgG_4_ antibodies from BPA, NBPA and NA subjects recognize mainly unfolded and sequential epitopes. IgG competition studies show that IgG specific for unfolded/sequential Bet v 1 epitopes is not inhibited by folded Bet v 1 and hence the latter seem to represent cryptic epitopes. IgG reactivity to Bet v 1 peptides did not correlate with IgG reactivity to the corresponding Mal d 1 peptides and therefore does not seem to be a result of primary sensitization to PR10 allergen‐containing food. Natural Bet v 1‐specific IgG antibodies inhibited IgE binding to Bet v 1 only poorly and could even enhance Bet v 1‐specific basophil activation.

**Conclusion:**

IgE and IgG antibodies from BPA patients and birch pollen‐exposed non‐sensitized subjects recognize different epitopes. These findings explain why natural allergen‐specific IgG do not protect against allergic symptoms and suggest that allergen‐specific IgE and IgG have different clonal origin.

## Graphical abstract

The recognition of conformational and sequential epitopes of Bet v 1 in birch pollen allergic patients (BPA), non‐birch pollen allergic patients (NBPA) and non‐allergic subjects (NA) was analysed. IgE antibodies from BPA patients recognized only conformational epitopes. IgG antibodies from all individuals recognized sequential and conformational epitopes. Bet v 1‐specific IgE‐ and IgG‐producing B cells may have different clonal origins.Abbreviations: BPA, Birch pollen allergic patients; ELISA, Enzyme‐linked immunoassay; IgE, Immunoglobulin E; IgG, Immunoglobulin G; IgM, Immunoglobulin M; NBPA, Allergic patients without birch pollen allergy; NA, Non‐allergic individuals.

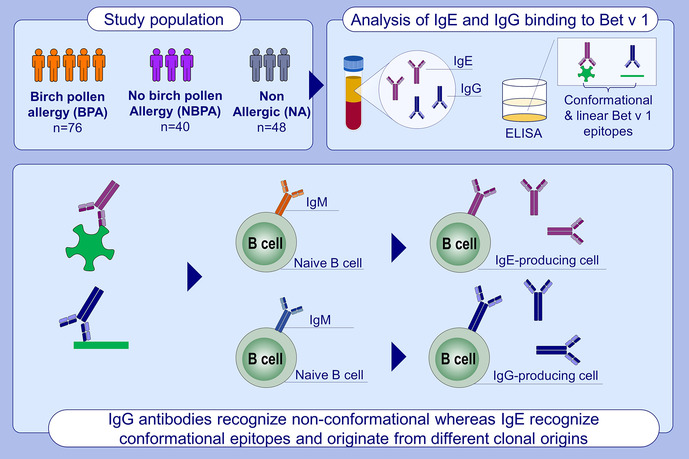

**Abbreviations**
aa: amino acid
ABTS: 2, 2′‐azino‐bis 3‐ethylbenzothiazoline‐6‐sulfonic acid
AD: atopic dermatitis
APC: antigen presenting cells
BPA: birch pollen allergic patients
BSA: bovine serum albumin
ELISA: enzyme‐linked immunoassay
f: female
F: fragment
h: hour
HBTU: 2‐(1H benzotriazole‐1‐yl)‐1,1,3,3‐tetramethyluronium hexafluorophosphate
HDM: house dust mite
HPLC: high‐pressure liquid chromatography
HRP: horseradish peroxidase
IgE: immunoglobulin E
IgG: immunoglobulin G
IPTG: isopropyl β‐D‐1‐thiogalactopyranoside
ISAAC: International study of asthma and allergies in childhood
ISU: ISAC standardized units
IVIG: intravenous immunoglobulin
LB: lysogeny broth
m: male
MALDI‐TOF: matrix assisted laser desorption – ionization ‐time of flight mass spectrometry
MeDALL: mechanisms of the development of allergy
MEM: minimum essential medium
min: minute
MW: molecular weight
NA: non‐allergic individuals
NBPA: allergic patients without birch pollen allergy
OAS: oral allergy syndrome
OD: optical density
P: peptide
PBS: phosphate buffered saline
PI: isoelectric point
r: recombinant
RBL: rat basophilic leukaemia
rpm: revolutions per minute
SCORAD: scoring atopic dermatitis
SD: standard deviation
SDS‐PAGE: sodium dodecyl sulphate–polyacrylamide gel electrophoresis

## 1 INTRODUCTION

IgE‐associated allergy is the most important immunologically‐mediated disease affecting more than 30% of the population.^1^ Allergic patients differ from non‐allergic subjects by their unique ability to produce IgE antibodies against mainly environmental antigens, termed allergens, which is regulated by a large variety of host and environmental factors.^2^, ^3^, ^4^, ^5^ Since the beginning of systematic allergen characterization by immunochemical and molecular biological methods, many if not most of the important allergen molecules have been characterized regarding primary sequence, structure, biological function and immunological features.^6^ With the availability of pure allergen molecules and defined epitopes thereof, it has become possible to investigate allergen‐specific IgE and T‐cell epitopes involved in allergic inflammation in sensitized patients.

However, relatively few studies have investigated the allergen‐specific immune response in allergic as compared to non‐allergic subjects.^7^ One seminal study has demonstrated that immune responses in healthy and allergic individuals are characterized by a fine balance between allergen‐specific T regulatory and T helper 2 cells and thus defined differences in cell types involved in the healthy and allergic immune response.^8^

However, it has been demonstrated that T‐cell epitope specificity and HLA recognition of the T‐cell epitopes are not critical for lymphokine production by allergen‐specific T‐cell clones,^9^ and thus, it seems that allergic and non‐allergic subjects recognize similar T‐cell epitopes on allergens.^10^

Similar as for T‐cell epitopes recognized by allergic and non‐allergic patients, little information is available regarding the specificity of IgE and natural IgG antibodies for allergen epitopes in allergic and non‐allergic subjects. For the major birch pollen allergen, Bet v 1, it has been demonstrated that allergen‐specific IgE antibodies recognize almost exclusively the folded Bet v 1 allergen but show no relevant reactivity to unfolded recombinant Bet v 1 fragments or to Bet v 1‐derived peptides lacking secondary structure.^11^, ^12^ However, the IgG antibody recognition to folded Bet v 1 versus unfolded fragments and peptides in allergic and non‐allergic subjects has not been studied. For house dust mite allergens, one study has shown that Der p 1‐specific IgE and IgG antibodies of allergic patients may react with similar recombinant allergen fragments^13^ whereas another study suggested that IgE of HDM allergic patients, similar as observed for Bet v 1, react mainly with conformational epitopes on HDM allergens.^10^

It is very important to understand whether there are differences between allergen epitopes recognized by IgE and IgG antibodies from allergic patients and non‐allergic subjects because some fundamental questions are connected to this topic. Regarding allergic patients, it is important to understand whether the clonal origin of the IgE and IgG responses is identical/similar or different as it may have implications for therapy, especially for allergen‐specific immunotherapy (AIT) because allergen‐specific IgG antibodies may influence allergen‐specific IgE recognition and IgE‐mediated allergic inflammation.^14^, ^15^, ^16^, ^17^ It is also of interest to know whether natural IgG antibodies in non‐sensitized subjects are directed to IgE epitopes recognized by allergic patients. For example, it is possible that natural allergen‐specific IgG antibodies recognizing IgE epitopes may protect against allergic sensitization when transmitted from mother to off‐spring.^18^ Furthermore, allergen‐specific IgG antibodies from non‐allergic subjects which block allergic patients IgE binding to allergens may be used for treatment of allergy by passive immunization, as it has been recently proposed for recombinant monoclonal allergen‐specific IgG antibodies.^19^, ^20^

In this study, we have investigated in detail the epitope specificity of Bet v 1‐specific IgE and IgG antibodies in allergic and non‐sensitized subjects taking advantage of the availability of the folded Bet v 1 allergen molecule and of recombinant unfolded Bet v 1 fragments and synthetic peptides spanning the complete Bet v 1 sequence to discriminate recognition of conformational versus non‐conformational epitopes by antibodies. Importantly, we have also studied the ability of Bet v 1‐specific IgG antibodies of non‐sensitized subjects to inhibit the binding of Bet v 1 allergic patients' IgE to Bet v 1 and Bet v 1‐induced basophil activation.

## 2 MATERIALS AND METHODS

### 2.1 Study subjects and subjects' sera

In one population, sera from 66 birch pollen allergic patients (BPA: 5–55 years old, 39 males and 27 females), 30 non‐birch‐sensitized allergic patients (NBPA: 10–30 years old, 16 males and 14 females) and 38 non‐allergic individuals (NA: 11–45 years old, 16 males and 22 females) were analysed in this study (Table 1 and Tables S1A, S1B, S1C). These participants were recruited in Moscow or in Yekaterinburg, Russia. Blood samples were obtained from these participants with approval of the respective Ethics Committees of the NRC Institute of Immunology FMBA of Russia, and of the Ural state Medical University, Russian Federation, after written informed consent had been obtained from the adult subjects or from the parents or the official representatives of children. In addition, sera from Austrian patients (BPA: *n* = 10, 27–68 years old, six males and four females; NBPA: *n* = 10, 12–73 years old, six males and four females; NA: *n* = 10, 26–51 years old, two males and eight females; Table S2) were analysed with approval of the Ethics committee of the Medical University of Vienna, Austria (EK1641/2014) after signed informed consent was obtained.

**TABLE 1:** Demographic and clinical characteristics of subjects.

Characteristics of subjects	Group 1 (BPA, *n* = 66)	Group 2 (NBPA, *n* = 30)	Group 3 (NA, *n* = 38)
*Gender*			
Male, no. (%)	39 (59%)	16 (53%)	16 (42%)
Female, no. (%)	27 (41%)	14 (47%)	22 (58%)
*Age (years)*			
Mean (±SD)	17.4 ± 10.9	13.6 ± 4.37	22.5 ± 9.3
Range	5–55	10–30	11–45
*Allergies, no. (%)*			
Trees	66 (100%)	0 (0%)	0 (0%)
Grass	33 (50%)	7 (23.3%)	0 (0%)
Weeds	25 (37.8%)	6 (20%)	0 (0%)
HDM	31 (46.9%)	15 (50%)	0 (0%)
Dog	28 (42.4%)	8 (26.7%)	0 (0%)
Cat	38 (58.6%)	15 (50%)	0 (0%)
Food allergy	13 (19.7%)	3 (10%)	0 (0%)
*Symptoms, no. (%)*			
Asthma	28 (42.4%)	12 (40%)	0 (0%)
Rhinoconjunctivitis	64 (97%)	26 (86.7%)	0 (0%)
Dermatitis	37 (56%)	13 (43.3%)	0 (0%)
OAS	40 (60.6%)	1 (3.33%)	0 (0%)

As the first step of recruitment, symptoms of allergy were recorded using the validated ISAAC questionnaire,^21^ as described.^22^, ^23^ Birch pollen allergy was confirmed for the group of birch pollen allergic patients (Group 1: BPA) or excluded for the group of allergic patients without birch pollen allergy (Group 2: NBPA) by a detailed case history, physical examination and skin prick testing according to guidelines,^24^ as well as by measuring birch pollen allergen‐specific IgE by ImmunoCAP technology (Thermofisher, Uppsala, Sweden) as described.^22^ Subjects having received allergen‐specific immunotherapy (AIT) have been excluded. The clinical diagnosis of allergic rhinitis was based on recommendations by the European Academy of Allergy and Clinical Immunology^25^ and ARIA guidelines.^26^ The diagnosis of asthma was performed according to guidelines of the Global Initiative for Asthma/Global Strategy for Asthma Management and Prevention.^27^

Atopic dermatitis was diagnosed based on international guidelines.^28^ Birch pollen‐related oral allergy syndrome was diagnosed based on a questionnaire approach.^29^ Total serum IgE was measured by Cormay Diagnostic Kit (Cormay Diagnostics) or by ImmunoCAP testing (Thermofisher). IgE sensitization to Bet v 1 in the BPA patients or lack of IgE sensitization to Bet v 1 in the NBPA patients in group 3 comprising non‐allergic subjects (Group 3: NA) was confirmed by ImmunoCAP ISAC testing (Thermofisher). Sensitization to more than 100 allergen molecules was excluded for the NA group also by ImmunoCAP ISAC testing. The cut‐off value for specific IgE in ImmunoCAP ISAC measurements was 0.3 ISU. The detailed demographic, clinical and serological characteristics of the subjects are presented in Table 1 and Tables S1A, S1B, S1C as well as in Table S2.

### 2.2 Expression and purification of recombinant Bet v 1 and recombinant Bet v 1 fragments

Bet v 1.0101 (GenBank: CAA33887), Mal d 1 (GenBank: AAD29671.1) and Bet v 1 fragments (F1: aa 1–74; F2: aa 75–160)^11^ were cloned into the NdeI and EcoRI restriction site of plasmid pET‐17b (Novagen). DNA sequences of the constructs were confirmed by sequence analysis (ATG: biosynthetics GmbH), and recombinant proteins were expressed with a 6x His‐tag at the C‐terminus in *E.coli* BL21 Gold (DE3) (Agilent Technologies).^30^ *E. coli* cell pellets containing soluble rBet v 1 or rMal d 1 were lysed in 50 mM NaH_2_PO_4_, 300 mM NaCl, 10 mM Imidazole, pH 8.0 for 2 h at 4°C whereas cell pellets containing rBet v 1 F1 and rBet v 1 F2 in inclusion bodies were lysed in 100 mM NaH_2_PO_4_, 8 M Urea, pH 8.0 buffer for 2 h at 4°C. Protein‐containing lysates were centrifuged for 20 min at 4°C, 10,000 rpm. Bet v 1‐, Mal d 1‐ and Bet v 1 fragment‐containing supernatants were purified by Ni‐NTA Agarose affinity chromatography (Qiagen). Buffer containing 50 mM NaH_2_PO_4_, 300 mM NaCl, 250 mM Imidazole, pH 8.0 was used for elution of recombinant Bet v 1 or Mal d 1 whereas recombinant fragments were eluted with 100 mM NaH_2_PO_4_, 8 M Urea, pH 4.5. Eluted samples were analysed by SDS‐PAGE, and thereafter, fractions containing recombinant proteins of more than 90% purity were pooled and dialyzed. Recombinant Bet v 1/Mal d 1 was dialyzed against 50 mM NaH_2_PO_4_, 300 mM NaCl, pH 8.0. Recombinant Bet v 1 F1 and F2 were dialyzed against 100 mM NaH_2_PO_4_, pH 4.5. The purified proteins were characterized by SDS‐PAGE, mass spectrometry, circular dichroism as well as by immunoblotting and ELISA for IgE reactivity as described.^30^

### 2.3 Synthetic Bet v 1‐derived peptides

Six non‐IgE‐reactive and non‐allergenic Bet v 1‐derived peptides described by Focke et al.^12^ (P1: aa 1–24; P2: aa 30–59; P3: aa 50–79; P6: aa 75–104; P4: aa 110–139; P5: aa 130–160; Table S3) were produced by chemical synthesis using 9‐fluorenylmethoxycarbonyl (Fmoc) amino acid protection and HBTU coupling on a peptide synthesizer (Liberty Blue, CEM Corporation). Peptides were purified to >90% purity by high‐pressure liquid chromatography (HPLC) (Dionex UltiMate 3000; Thermo Fisher Scientific), and their molecular weights were checked by MALDI‐TOF mass spectrometry (Microflex, Bruker). For comparing peptide‐specific IgG reactivity to Bet v 1 and Mal d 1 by micro‐array analysis, seven Bet v 1‐derived peptides as described in^31^ and the corresponding Mal d 1 peptides were prepared and characterized as described above.

### 2.4 Measurement of IgE and IgG antibody levels specific for Bet v 1, Bet v 1 fragments and Bet v 1‐derived peptides

Specific IgE and IgG antibody levels in sera were determined by ELISA. ELISA plates (Greiner bio‐one) were coated in triplicates with equimolar amounts of rBet v 1 (2 μg/mL), rBet v 1 F1 or F2 (1 μg/mL), an equimolar mix of F1 + F2 or Bet v 1 peptides (370 ng/mL) in 100 mM carbonate buffer, pH 9.6 (100 μL/well) overnight at 4°C. Coated antigen concentrations were determined in pilot ELISA experiments to ensure antigen excess over antibodies. Plates were then washed three times with PBS 0.05% Tween 20 (200 μL/well) and then blocked with 2%BSA in PBS 0.05% Tween 20 overnight at 4°C (100 μL/well). Sera and antibodies were diluted in 0.5% BSA in PBS 0.05% Tween 20. Plates were incubated with sera diluted 1:10 for measurement of IgE levels and 1:100 for measurement of IgG levels (overnight at 4°C) (100 μL/well). Plates were then washed five times with PBS 0.05% Tween 20 (200 μL/well). For IgE detection, plates were incubated with goat anti‐human IgE‐HRP antibody (KPL) diluted 1:2500 (100 μL/well) for 1 h at 37°C and 1 h at 4°C. For IgG detection, plates were first incubated with AffiniPure Rabbit Anti‐Human IgG (Jackson ImmunoResearch Laboratories) diluted 1:1000 (100 μL/well) overnight at 4°C. Thereafter, plates were washed 5 times with PBS 0.05% Tween 20 (200 μL/well) and then incubated with Anti‐Rabbit IgG, HRP from donkey (GE Healthcare GmbH) (1:2000) (100 μL/well) for 1 h at 37°C and 1 h at 4°C. Finally, plates were washed four times as described above and colorimetric detection was done with 2,2′‐azino‐bis 3‐ethylbenzothiazoline‐6‐sulphonic acid (ABTS) (Sigma‐Aldrich) solution in citric acid buffer (100 μL/well). Optical densities (OD) were measured using an ELISA reader (Thermo Scientific, Multiskan, GO) at 405/490 nm wavelength. To harmonize and calibrate regarding plate‐to‐plate variabilities, experiments were performed so that a calibration serum was included on each of the plates. Triplicate measurements were performed, and then, the mean of the triplicates was calculated. Cut‐off values were determined as the highest negative control mean + 2 SD. The results are displayed as median and interquartile range. For control purposes, buffer instead of serum was applied and tested.

### 2.5 Micro‐array‐based measurement of IgE and IgG reactivity to Bet v 1, Mal d 1 and Bet v 1‐ and Mal d 1‐derived peptides

Bet v 1, Mal d 1, the seven Bet v 1‐derived peptides, Bet v 1 p1–p7^31^ and the corresponding seven Mal d 1‐derived peptides, Mal d 1 p1–p7 were spotted onto a glass slide in triplicates as described.^32^ Specific IgE levels and IgG levels were measured using fluorescence‐labelled anti‐human IgE or IgG antibodies conjugated with fluorophore (DyLight™ 550‐2xPEG NHS Ester) for 30 min at RT.^32^, ^33^ The slides were washed, dried and analysed with a Tecan PowerScanner™ and are expressed as ISU‐IgE or ISU‐IgG, respectively.

### 2.6 ELISA IgG competition experiments

ELISA plates (Nunc MaxiSorp 96‐well flat bottom, Thermo Fisher Scientific) were coated in duplicates with 1 μg/mL rBet v 1 or an equimolar mixture of F1 + F2 in 100 mM carbonate buffer, pH 9.6 (100 μL/well) for 5 h at RT. Plates were washed three times with PBS 0.05% Tween 20 (200 μL/well) and then blocked with 2%BSA in PBS 0.05% Tween 20 overnight at 4°C (100 μL/well). Sera from birch pollen allergic patients and non‐allergic subjects diluted 1:100 were incubated overnight either with 100 μg/mL BSA, Bet v 1 or with a mix of 50 μg/mL of each of the Bet v 1 fragments overnight at 4°C and added (100 μL/well). As negative control, 100 μg/mL BSA, Bet v 1 or the mix of 50 μg/mL Bet v 1 fragments were incubated with buffer alone overnight at 4°C and added (100 μL/well). On the next day, plates were washed four times with PBS 0.05% Tween 20 (200 μL/well) and incubated with AffiniPure Rabbit Anti‐Human IgG (Jackson ImmunoResearch Laboratories) diluted 1:1000 (100 μL/well) overnight at 4°C. Next day, plates were washed five times with PBS 0.05% Tween 20 (200 μL/well) and then incubated with anti‐Rabbit IgG, HRP from donkey (GE Healthcare GmbH) (1:2000) (100 μL/well) for 1 h at 37°C and 1 h at 4°C. In the final step, plates were washed four times as described above and colorimetric detection was performed with ABTS (Sigma‐Aldrich) solution in citric acid buffer (100 μL/well). Optical densities (OD) were measured using an ELISA reader (Infinite® F50 Plus) at 405/490 nm wavelength. The results are expressed as average (less than 5% deviation of values) OD values corresponding to bound IgG of duplicate measurements with median, upper and lower quartiles. Percentages inhibitions were calculated as described in 2.8.

### 2.7 Determination of Bet v 1‐ and Bet v 1 fragment‐specific IgG_1_ and IgG_4_ antibody levels

Determination of IgG_1_ and IgG_4_ antibodies specific for Bet v 1, Bet v 1 F1 and F2 was performed for birch pollen allergic patients and non‐allergic subjects. ELISA plates (Greiner bio‐one) were coated in triplicates with 2 μg/mL of Bet v 1 and with 1 μg/mL of each Bet v 1 fragment for overnight at 4°C as described above. After three washes with PBS 0.05% Tween 20 (200 μL/well), nonspecific binding sites were blocked with 2%BSA in PBS 0.05% Tween 20 (100 μL/well, overnight at 4°C). Plates were incubated with sera diluted 1:100 in 0.5% BSA in PBS 0.05% Tween 20, overnight at 4°C (100 μL/well). Thereafter, plates were washed five times as described above and then incubated overnight at 4°C with a mixture of biotin‐labelled mouse anti‐human IgG_1_ or IgG_4_ (Thermo Fisher Scientific) and horseradish peroxidase (HRP)‐conjugated streptavidin (Bio‐Rad) (100 μL/well). Both components were diluted 1:2500 in 0.5% BSA in PBS/0.05% Tween 20 and mixed together prior incubation. After four washes (PBS 0.05% Tween 20, 200 μL/well), colour reaction was performed by adding ABTS substrate solution (Sigma‐Aldrich) in citric acid buffer (100 μL/well) and optical densities (OD) were measured at a wavelength of 405/490 nm in an ELISA reader (Thermo Scientific Multiskan, GO). To harmonize and calibrate regarding plate‐to‐plate variabilities, experiments were performed so that a calibration serum was included on all of the plates. The means of triplicate measurements were calculated. Cut‐off values were determined as the highest negative control mean + 2 SD of the negative control readings. The results are displayed as median and interquartile range. The sensitivities of the detection systems for measuring allergen‐specific IgG_1_ and IgG_4_ antibodies were evaluated with human monoclonal allergen‐specific IgG_1_ and IgG_4_ antibodies, respectively, and found to be 2.7‐fold more sensitive for IgG_4._ ^34^

### 2.8 ELISA IgE competition experiments

ELISA plates (Greiner bio‐one) were coated in triplicates with Bet v 1 (2 μg/mL), in 100 mM carbonate buffer, pH 9.6 (100 μL/well) overnight at 4°C. Plates were washed three times with PBS 0.05% Tween 20 (200 μL/well) and then blocked with 2%BSA PBS 0.05% Tween 20 (100 μL/well) overnight at 4°C. Plate‐bound Bet v 1 was incubated with sera from non‐allergic subjects diluted 1:2 in 0.5% BSA in PBS 0.05% Tween 20 for 2 h at 37°C and 1 h at 4°C (100 μL/well). After three washes (PBS 0.05% Tween 20) (200 μL/well), plates were incubated with sera from Bet v 1 allergic patients (1:5 diluted in 0.5% BSA in PBS 0.05% Tween 20) for overnight at 4°C (100 μL/well). Plates were washed five times (PBS 0.05% Tween 20) (200 μL/well), and bound IgE antibodies were detected with goat anti‐human IgE‐HRP antibody (KPL) diluted 1:2500 in PBS/ 0.05% Tween 20/0.5% BSA by first incubating the plates at 37°C for 1 h and then at 4°C for additional 1 h. After 5 washes (PBS 0.05% Tween 20) (200 μL/well), colour reaction was determined^34^ and optical density was measured using an ELISA reader (Thermo Scientific Multiskan, GO) at 405/490 nm. The percentage inhibition of IgE binding was calculated as previously described^35^: percentage of inhibition = 100 – OD_with inhibitor_/OD_without inhibitor_ × 100. The results are expressed as mean % of inhibition of triplicate measurements with median, upper and lower quartiles.

### 2.9 RBL degranulation assays

Rat basophilic leukaemia (RBL) cells (RSATL8) expressing the human high‐affinity IgE receptor, FcεRI were grown in MEM medium (Gibco, Gibco, Thermo Fischer Scientific) supplemented with 10% FBS, 2 mM L‐glutamine, 100 units/mL penicillin, 100 μg/mL streptomycin, 10 mg/mL geneticin and 10 mg/mL Hygromycin B (Thermo Fisher Scientific). Cells were then seeded in duplicates (2 × 10^5^ cells/well) (100 μL/well) into 96‐well flat bottom cell culture plate (Corning, Thermo Fischer Scientific), loaded with sera from birch pollen allergic patients 1:10 diluted in MEM medium and cultured overnight at 37°C in 5% CO_2_. Cells only with MEM medium were used as control. Thereafter, cells were washed twice with Tyrode's buffer (Sigma‐Aldrich) and IgE‐loaded cells were stimulated with different concentrations of Bet v 1 (1000, 100, 10 ng/mL) or with a pre‐incubated mix of sera from non‐allergic subjects and allergen for 1 h at 37°C. In order to study the effects of IgG‐containing serum on Bet v 1‐induced basophil activation in the very same allergic patient, cells loaded with IgE from the patients were stimulated with different concentrations of Bet v 1 (1000, 100, 10, 1 ng/mL) or with a pre‐incubated mix of heat‐inactivated serum from the very same patient and the aforementioned allergen concentrations for 1 h at 37°C. Cells were washed three times with 200 μL/well washing buffer (i.e. Tyrode's salts, 0.02 M NaHCO_3_, 1% w/v BSA in H_2_O, pH 7.2), and β‐hexosaminidase release was detected in the cell supernatants with the addition of 4‐Nitrophenyl‐N‐acetyl‐β‐ D‐glucosaminide as described.^36^ For determination of 100% mediator release, cells were lysed with 10% v/v Triton X‐100 (Merck Millipore). Plates were read on an ELISA reader (Thermo Scientific Multiskan, GO) at 405/490 nm. The results were calculated as mean of duplicates and are displayed as the percentages of total β‐hexosaminidase release.

### 2.10 Statistical methods

Specific IgE and IgG antibody levels for Bet v 1 and Bet v 1‐specific fragments as well as Bet v 1–derived peptides and specific IgG_1_ and IgG_4_ antibody levels for Bet v 1 and Bet v 1‐specific fragments were submitted to a distribution analyses after subtraction of the group means to obtain residuals. All these OD values deviated from normality as expected; however, a log‐normal distribution fitted the data well. Therefore, data were analysed by a General Linear Model with a log‐transformation. A mixed model was applied with the within subject factor antigen (Bet v 1, F1, and F2 or peptides P1–P6) and the between subject factor group (BPA, NBPA and NA). Specific hypotheses were tested, except for peptides, by linear contrasts with *p* values corrected by the Bonferroni–Holm method. Due to the exploratory nature of the comparisons of peptides, antibody levels were submitted to Tukey's HSD post‐hoc tests. In graphical presentations, the data are summarized as dot plots with medians and interquartile ranges. Pearson correlations between transformed IgE and IgG as well as IgG_1_ and IgG_4_ levels in BPA individuals were computed, and significance of the linear relationship was tested by a two‐sided t‐test. For correlations, only IgE levels above the cut‐off (OD = 0.42) were included. The sample size was sufficient to detect a linear relationship with a coefficient of determination exceeding about 15%. A coefficient exceeding ±0.5 was considered reflecting a strong relationship and those between 0.4 and 0.49 as moderate.

In general, *p* values below .05 were considered statistically significant. Statistical analyses were performed using Stata 17.0 (StataCorp), and graphs were produced with GraphPad Prism 8.0 (GraphPad Software).

## 3 RESULTS

### 3.1 Characterization of study subjects

Table 1 provides a summary of the demographic and clinical characteristics of the Russian subjects investigated in this study whereas Table S1C show detailed demographic, clinical and serological parameters for each of the studied subjects. In total, hundred and thirty‐four subjects (71 males and 63 females) were recruited and allocated to three groups (Table 1). Group 1 (BPA) included 66 patients (39 males, 27 females; age range: 5–55 years; mean age: 17.4 years) suffering from different symptoms of birch pollen allergy. Sixty‐four of these patients suffered from birch rhinoconjunctivitis, 40 from oral allergy syndrome (OAS), 37 from atopic dermatitis (AD) and 28 from asthma related to birch pollen exposure. Thirteen patients had other manifestations of food allergies, not OAS: urticaria, angioedema, anaphylaxis, gastroenteritis, not related to cross‐sensitization to tree pollen allergens (Table 1). Patients from Group 1 were sensitized also to other allergen sources as detected by skin prick testing (e.g. trees: *n* = 66; cat: *n* = 38; grass: *n* = 33; house dust mites (HDM): *n* = 31; dog: *n* = 28; weeds: *n* = 25; Table 1 and Table S1A).

Group 2 (NBPA) consisted of 30 allergic patients without birch pollen sensitization (16 males, 14 females; age range: 10–30 years; mean age: 13.6 years). Among the NBPA patients, sensitizations determined by SPT were as follows: Cat: *n* = 15; HDM: *n* = 15; dog: *n* = 8; grass: *n* = 7: weed: *n* = 6 (Table 1 and Table S1B).

NBPA patients showed rhinoconjunctivitis as the most common allergic symptom (*n* = 26), followed by atopic dermatitis (*n* = 13), asthma (*n* = 12) and OAS (*n* = 1). None of the BPA and NBPA patients had ever received any type of AIT.

Group 3 (NA) included 38 non‐allergic individuals (16 males, 22 females; age range: 11–45 years; mean age 22.5 years). They did not report any allergic symptoms, had a negative SPT for birch pollen extract and/or mixed tree pollen extracts and no sensitizations were recorded in this group by ImmunoCAP ISAC testing and/or SPT (Table 1 and Table S1C). Table 1 shows that there was a comparable sex and age distribution among the BPA, NBPA and NA subjects who were compared for IgE, IgG and IgG subclass reactivity to Bet v 1, rBet v 1 fragments and Bet v 1 peptides (BPA: *n* = 66; 39 males, 27 females; age range: 5–55 years; mean age 17.4 years; NBPA: *n* = 30;16 males, 14 females; age range: 10–30 years; mean age 13.6 years; NA: *n* = 38; 16 males, 22 females; age range: 11–45 years; mean age 22.5 years). Certain experiments as indicated in the results were repeated or newly performed with BPA, NBPA and NA subjects from Austria (Table S2). One NBPA patient in this group was sensitized to ash and one to plane tree.

### 3.2 IgE antibodies from BPA patients react with complete and folded Bet v 1 but not with sequential peptide epitopes

**FIGURE 1:**
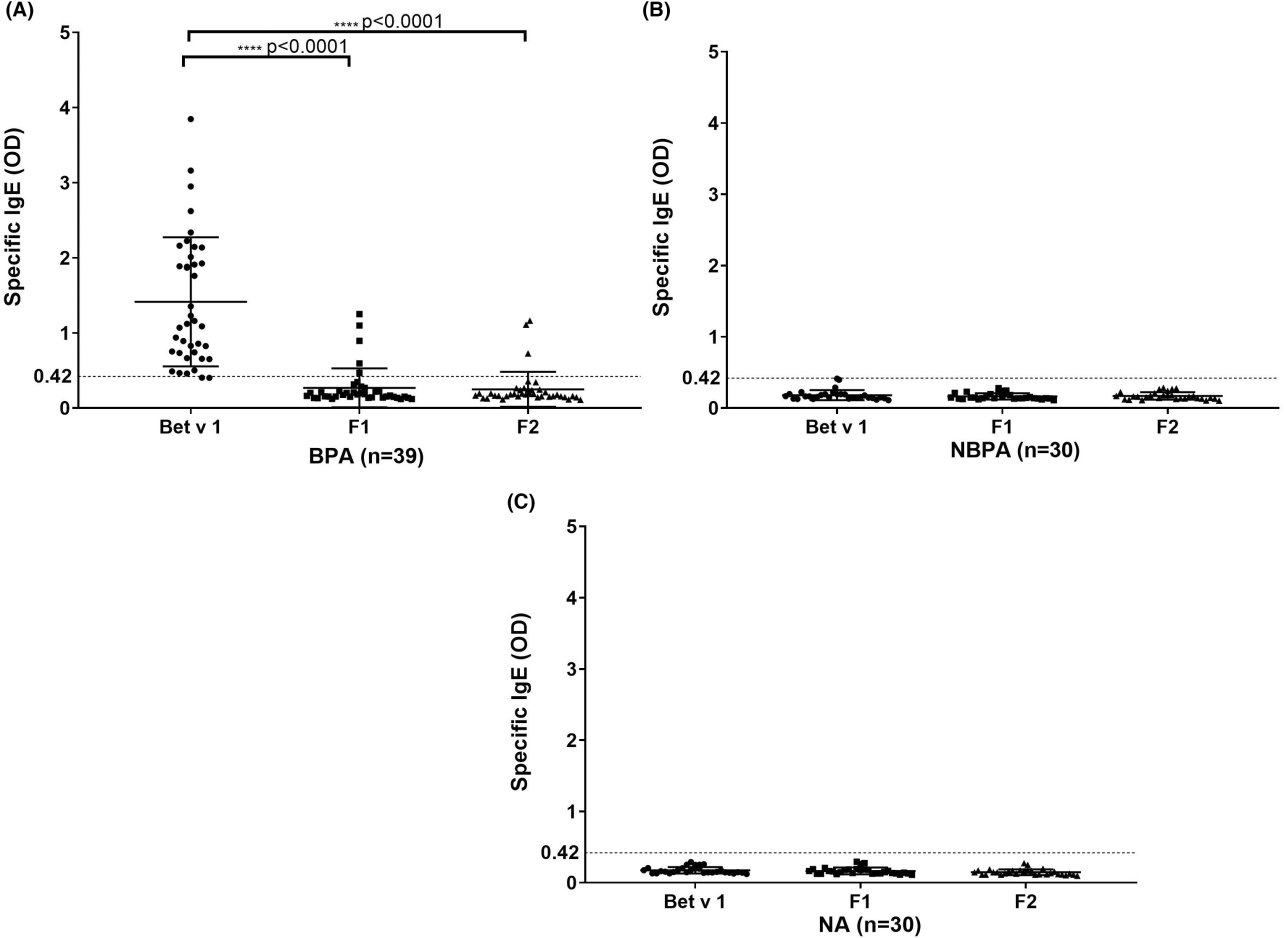
IgE reactivity to rBet v 1, F1 and F2 demonstrated by ELISA. Shown are IgE levels (y‐axes: OD values, median and interquartile range) in (A) sera from birch pollen allergic patients (BPA), (B) allergic patients without birch pollen allergy (NBPA) and (C) non‐allergic individuals (NA) specific for Bet v 1, F1, or F2 (x‐axes). The buffer control was subtracted from the data, and the cut‐offs are represented by horizontal dashed lines. Statistically significant differences between specific IgE levels to Bet v 1 and F1 or F2 are indicated (****p < .0001).

Our first set of experiments confirms earlier results^11^, ^12^ that IgE antibodies from the BPA patients investigated in our study reacted with complete and folded Bet v 1 (Figure 1A) but only very few patients showed IgE reactivity to unfolded rBet v 1 fragments F1 (Table S4, BPA 11, 13, 15, 23, 24) and F2 (Table S4, BPA: 13, 23, 24; Figure 1A) or with sequential Bet v 1 peptides (Figure S1A and Table S4, P2: BPA 11, 13, 23; P3: BPA 23; P5: BPA 24). Accordingly, specific IgE levels to Bet v 1 were significantly higher than specific IgE levels to F1 and F2 in the BPA group (*p* < .0001). No IgE reactivity to Bet v 1, rBet v 1 fragments or Bet v 1 peptides was found in NBPA and NA subjects (Figure 1B,C).

### 3.3 IgG antibodies from BPA, NBPA and NA subjects recognize unfolded rBet v 1 fragments and sequential Bet v 1 epitopes

**FIGURE 2:**
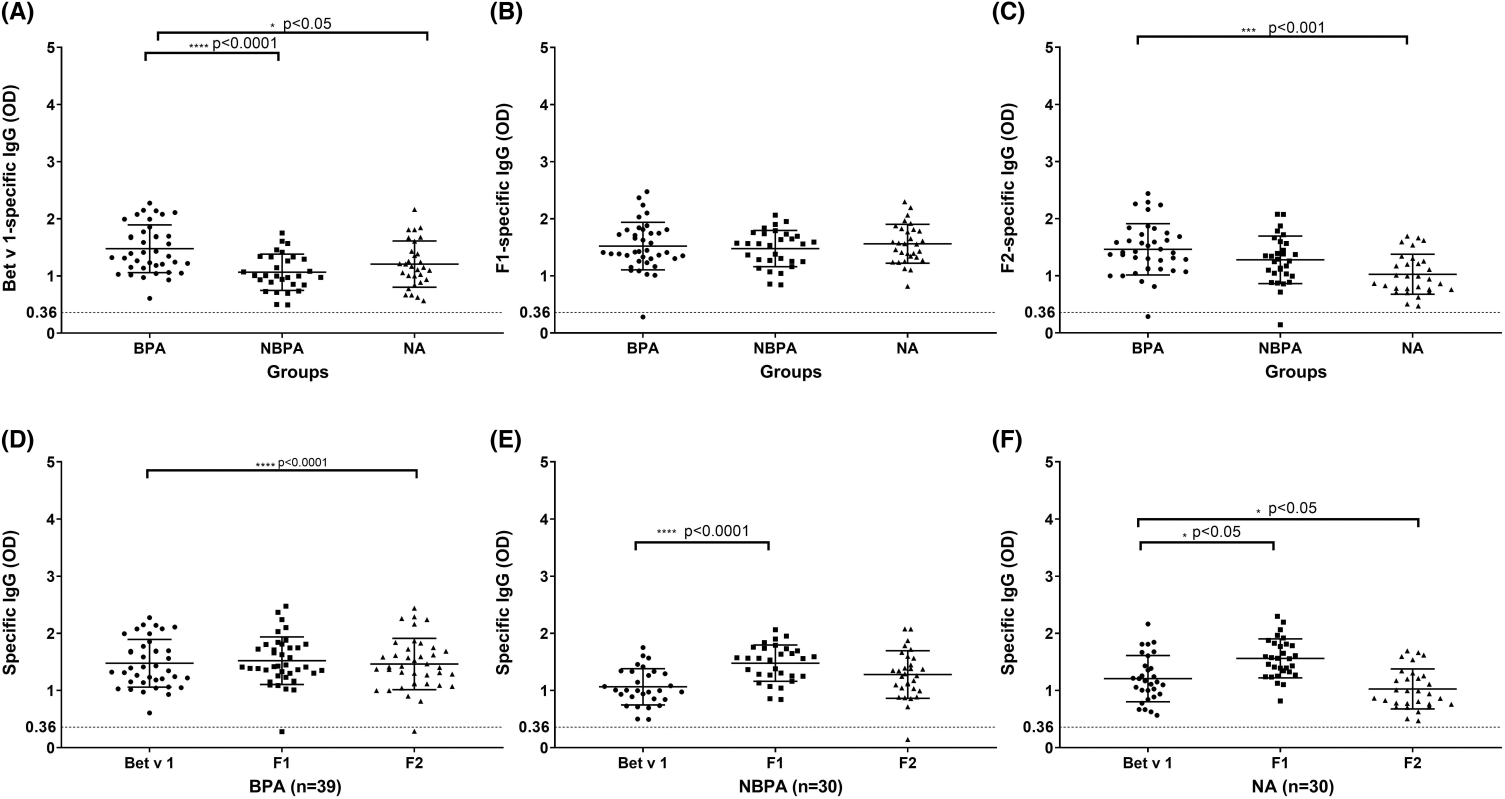
IgG levels specific for Bet v 1, F1 and F2 in BPA, NBPA and NA subjects. Shown are (A) rBet v 1‐, (B) F1‐ and (C) F2‐specific IgG levels (y‐axes: OD values, median and interquartile range) in sera from birch pollen allergic patients (BPA), allergic patients without birch pollen allergy (NBPA) and non‐allergic individuals (NA) (x‐axes). Panels (D‐F) compare Bet v 1‐, F1‐ and F2‐specific IgG levels within the groups (BPA, NBPA and NA). Cut‐off levels for a positive reaction are indicated by dashed horizontal lines. Significant differences between specific IgG levels to Bet v 1, F1 or F2 (D‐F) and comparisons between BPA versus NBPA and BPA and NA (A‐C) are indicated (****p < .0001; ***p < .001; *p < .05).

In contrast to IgE antibodies, IgG antibodies from BPA patients reacted not only with complete folded Bet v 1 (Figure 2A) but also with unfolded Bet v 1 fragments (F1: Figure 2B; F2: Figure 2C and Table S5). All but one BPA patient showed IgG reactivity to F1, and the same result was obtained for F2 (Figure 2D). IgG levels to F1 in BPA patients were comparable to Bet v 1‐specific IgG and for F2 significantly higher than for Bet v 1 (Figure 2D) and the sum of mean fragment‐specific IgG exceeded that of Bet v 1‐specific IgG (Table S5).

Interestingly, Bet v 1‐specific IgG levels were significantly higher in BPA patients than in the NBPA (*p* < .0001) and NA (*p* < .05) groups (Figure 2A). F1‐specific IgG levels were higher in the BPA group than in the NBPA and NA groups (Table S5). Likewise F2‐specific IgG levels were higher in the BPA group than in the NBPA group and this difference was significant when BPA and NA subjects were compared (Figure 2B,C and Table S5).

F1‐specific IgG levels were significantly higher than Bet v 1‐specific IgG levels in the NBPA group (*p* < .0001; Figure 2E) and in the NA group (*p* < .05; Figure 2F). By contrast, Bet v 1‐specific IgG levels were significantly higher than F2‐specific IgG levels in the NA group (*p* < .05; Figure 2F). Almost all sera from subjects from the BPA, NBPA and NA groups showed IgG reactivity to Bet v 1‐derived peptides (Figure S2 and Table S5).

P3‐specific IgG levels were higher than IgG levels against the other tested peptides in BPA patients (Figure S2A and Table S5). In BPA patients, P6‐specific IgG levels were significantly lower than those against all the other peptides (Figure S2A).

P5‐specific IgG levels were higher than IgG levels against the other tested peptides in NBPA and NA subjects (Figure S2B,C and Table S5). In NBPA and NA subjects, P6‐ and P3‐specific IgG levels were lower than those against other peptides.

### 3.4 IgG inhibition experiments indicate that unfolded IgG epitopes are cryptic

In order to study possible differences of IgG epitopes present on folded Bet v 1 and unfolded Bet v 1 fragments, IgG inhibition studies were performed in BPA, NBPA and NA subjects shown in Table S2. Results obtained document that IgG binding to folded Bet v 1 containing mainly conformational epitopes is inhibited best by folded Bet v 1 but not by the mix of unfolded F1 and F2 whereas IgG binding to the mix of unfolded F1 and F2 is best inhibited by F1 + F2 and not by folded Bet v 1 (Figures S3). Pre‐incubation of sera with Bet v 1 even seemed to enhance the IgE binding to the fragments probably due to formation of immune complexes. This result was obtained for the study population shown in Table S2 including BPA, NBPA and NA subjects (Table S2 and Figure S3) and also for the individual groups (BPA, NBPA and NA, data not shown). Thus, IgG reactivity to unfolded Bet v 1 fragments may in part be directed to cryptic epitopes which become available only after fragmentation or denaturation of the allergen whereas the IgG epitopes recognized on folded Bet v 1 are exposed on the surface of the folded allergen.

### 3.5 Bet v 1‐specific IgG_1_ and IgG_4_ is higher in BPA patients than in NA subjects and show different epitope recognition

**FIGURE 3:**
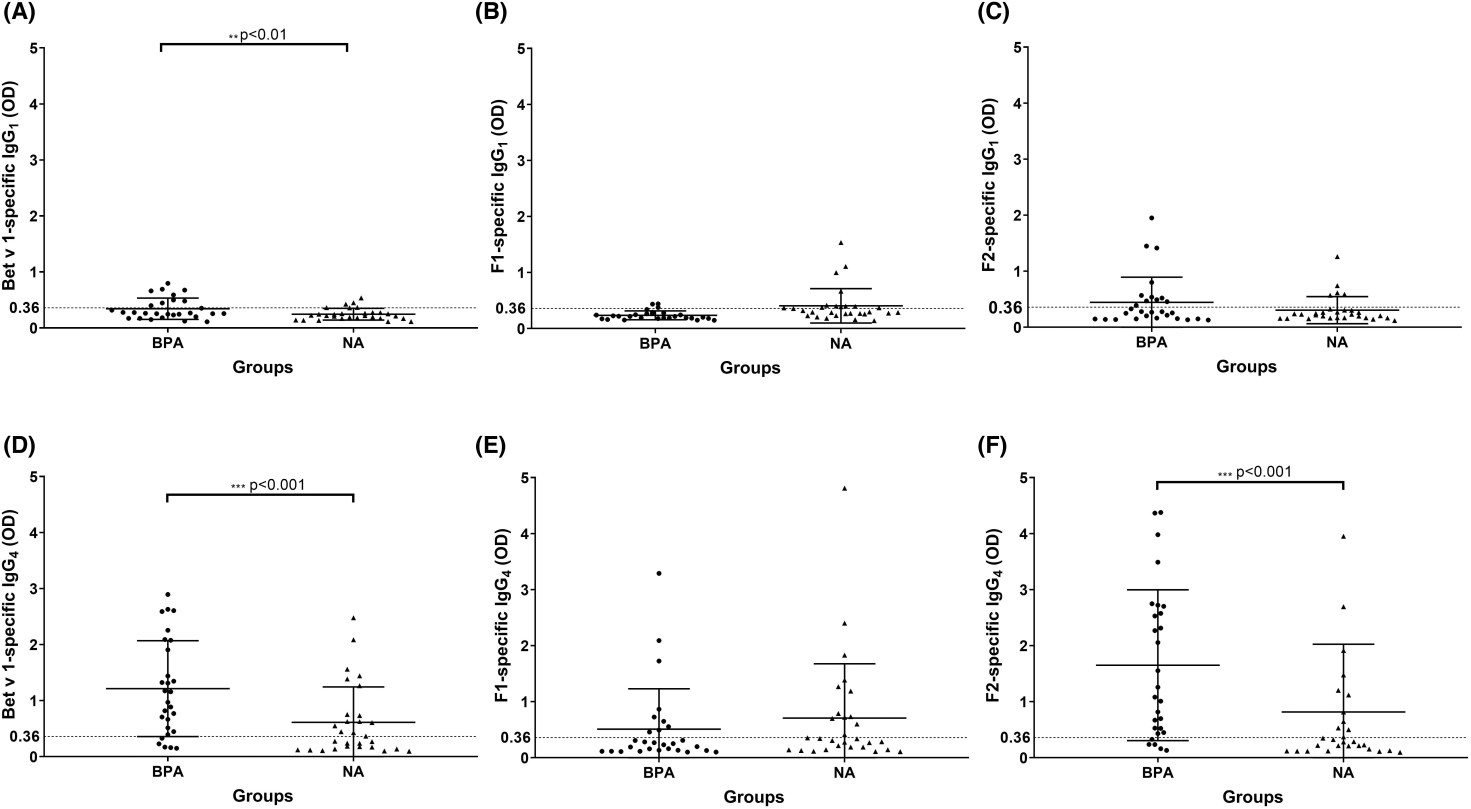
Specific IgG_1_ (A–C) and IgG_4_ levels (D–F) specific for rBet v 1, F1 and F2 (y‐axes: OD values, median and interquartile range) in sera from birch pollen allergic patients (BPA) and non‐allergic individuals (NA) (x‐axes). Statistically significant differences regarding Bet v 1‐specific IgG_1_/IgG_4_ levels between BPA and NA groups are indicated (***p < .001; **p < .01).

Figure 3A–F compares IgG_1_ and IgG_4_ antibody levels in BPA and NA subjects specific for Bet v 1 and for Bet v 1 fragments, respectively. We found that BPA patients have significantly higher IgG_1_ and IgG_4_ levels to Bet v 1 than NA subjects (*p* < .01 and *p* < .001, respectively; Figure 3A,D and Table S6). Also, F2‐specific IgG_1_ and IgG_4_ levels were higher in the BPA group as compared to the NA group and this difference was significant for IgG_4_ (*p* < .001; Figure 3F and Table S6). Interestingly, F1‐specific IgG_1_ and IgG_4_ antibody levels seemed to be slightly higher in the NA group than in the BPA group. However, IgG_1_ levels specific for F1 were generally very low (Figure 3B,E).

Of note, cumulative F1‐ and F2‐specific IgG_1_ and IgG_4_ levels were much higher than Bet v 1‐specific IgG_1_ and IgG_4_ levels in the BPA and NA group, respectively, indicating that IgG_1_ and IgG_4_ antibodies recognize preferentially non‐conformational Bet v 1 epitopes (Table S6). It is important to note here that the sensitivity of the IgG_4_ detection system used was approximately threefold as sensitive as that for IgG_1_ as determined with monoclonal allergen‐specific IgG_1_ and IgG_4_ antibodies (data not shown) but we have only compared antibody levels within a given subclass.

Figure S4 confirms that IgG levels specific for F1 + F2 were significantly higher than IgG levels specific for folded Bet v 1 in BPA and NA subjects (*p* < .05) when tested by ELISA under conditions of antigen excess. F1 + F2‐specific IgG levels were also higher for NBPA patients than IgG levels specific for folded Bet v 1 but this difference was not significant (Figure S4).

### 3.6 Levels of Bet v 1‐ and Bet v 1 fragment‐specific IgG, IgG_1_ and IgG_4_ antibodies do not correlate with specific IgE in birch pollen allergic patients

**FIGURE 4:**
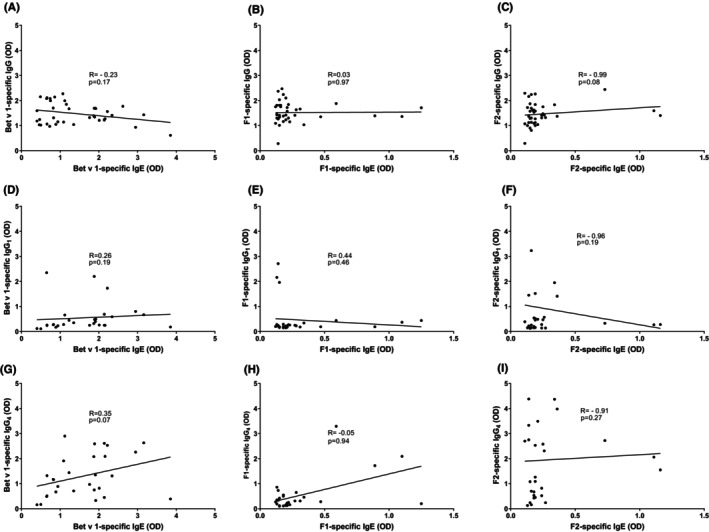
Correlation between IgE (x‐axes) and IgG, IgG1 and IgG4 levels (y‐axes) specific for Bet v 1, F1 and F2 (A‐I) in BPA patients.

In Figure 4A–I, we present a correlation analysis of the IgE levels to Bet v 1 with Bet v 1‐specific IgG, IgG_1_ and IgG_4_ antibodies in BPA patients. This analysis was performed to investigate whether allergen and fragment‐specific IgE and IgG responses in BPA patients are associated which would provide a hint for a clonal relationship and/or comparable activity of IgE and IgG‐producing B‐cell clones in the patients. No significant correlations were found between Bet v 1‐specific IgE production and Bet v 1‐specific IgG, IgG_1_ or IgG_4_ production (Figure 4A,D,G). The lack of association became even much stronger at the level of non‐conformational epitopes presented on F1 and F2. The majority of BPA patients lacked F1‐ and F2‐specific IgE (Table S4) but exhibited F1‐ and F2‐specific IgG as well as IgG_1_ and IgG_4_ responses (Tables S4, S5). Accordingly, there was no correlation at all between F1‐ and F2‐specific IgE and IgG, IgG_1_ or IgG_4_ responses (Figure 4B,C,E,F,H,I).

### 3.7 Levels of IgG specific for Mal d 1, Bet v 1 and corresponding peptides thereof show no relevant correlation

Figure S5 presents the correlations between the IgG reactivity to Bet v 1 and Mal d 1 as well as of the seven corresponding Bet v 1 and Mal d 1‐derived peptides as determined by micro‐array analysis for the population in Table S2. We found no relevant correlations between Bet v 1 and Mal d 1‐specific IgG (Figure S5, upper left corner). Likewise no relevant correlation was found between IgG reactivity to Bet v 1 peptides p1‐p and Mal d 1 peptides p1‐p5. There was some association of Mal d 1 p6, p7 and Bet v 1 p6, p7 IgG reactivity but IgG levels for p6 and p 7 were mostly higher for Bet v 1 (Figure S5).

### 3.8 Poor and varying inhibition of allergic patients IgE binding to Bet v 1 by IgG antibodies from non‐allergic individuals

**FIGURE 5:**
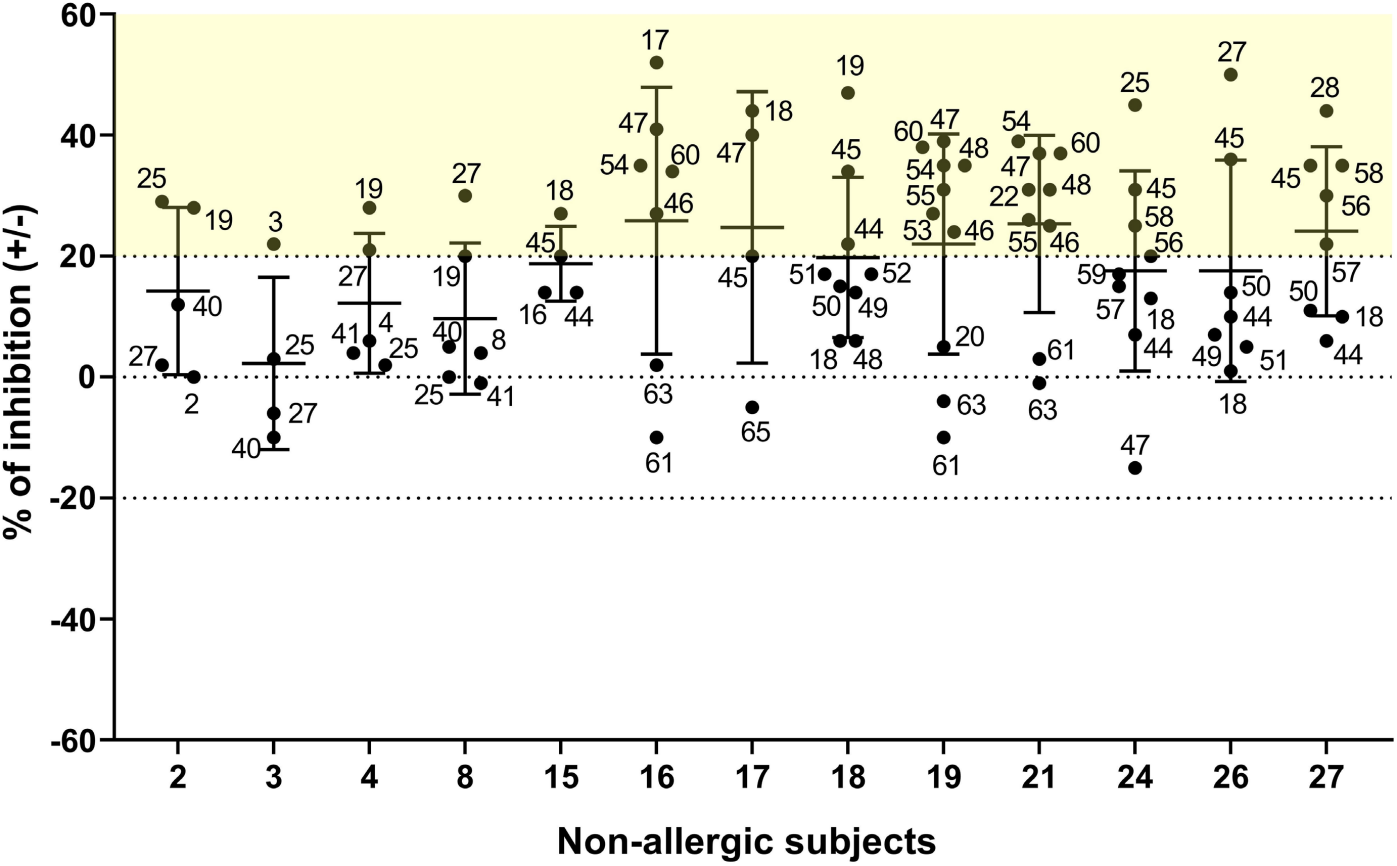
Effects of serum antibodies from non‐allergic subjects on the binding of IgE from birch pollen allergic subjects to Bet v 1. Shown are percentages of inhibition of IgE binding to Bet v 1 (median, lower and upper quartile) of birch pollen allergic patients (indicated by dots and the corresponding patients number) by sera from non‐allergic subjects (x‐axis: numbers of non‐allergic subjects).

We initially had hypothesized that non‐allergic subjects may possess IgG antibodies which may block IgE recognition of Bet v 1 in BPA patients. To investigate this assumption, we pre‐incubated ELISA plate‐bound Bet v 1 with serum antibodies from non‐allergic subjects (Figure 5: x‐axis) and measured the percentage inhibition of birch pollen allergic patients IgE binding (Figure 5: y‐axis) to Bet v 1. We found that for the majority of BPA patients an inhibition of equal or less than 20% was achieved whereas inhibition of IgE binding was of up to 50% was rare (Figure 5 and Table S7). Remarkably, the inhibition of IgE binding showed considerable variation depending on what NA serum was used for a particular BPA patient (Figure 5 and Table S7) indicating considerable diversity regarding epitope recognitions and/or avidity of blocking antibodies. The ability of serum of a given NA patient to inhibit IgE binding was not consistently associated with the levels of Bet v 1‐specific IgG (Table S7). Interestingly, we found that sera from certain NA subjects could inhibit but also moderately enhance allergic patients' IgE binding to Bet v 1 depending on what allergic patient was tested (Figure 5 and Table S7).

### 3.9 Serum antibodies from non‐allergic individuals can inhibit and enhance Bet v 1‐specific basophil activation in BPA patients

**FIGURE 6:**
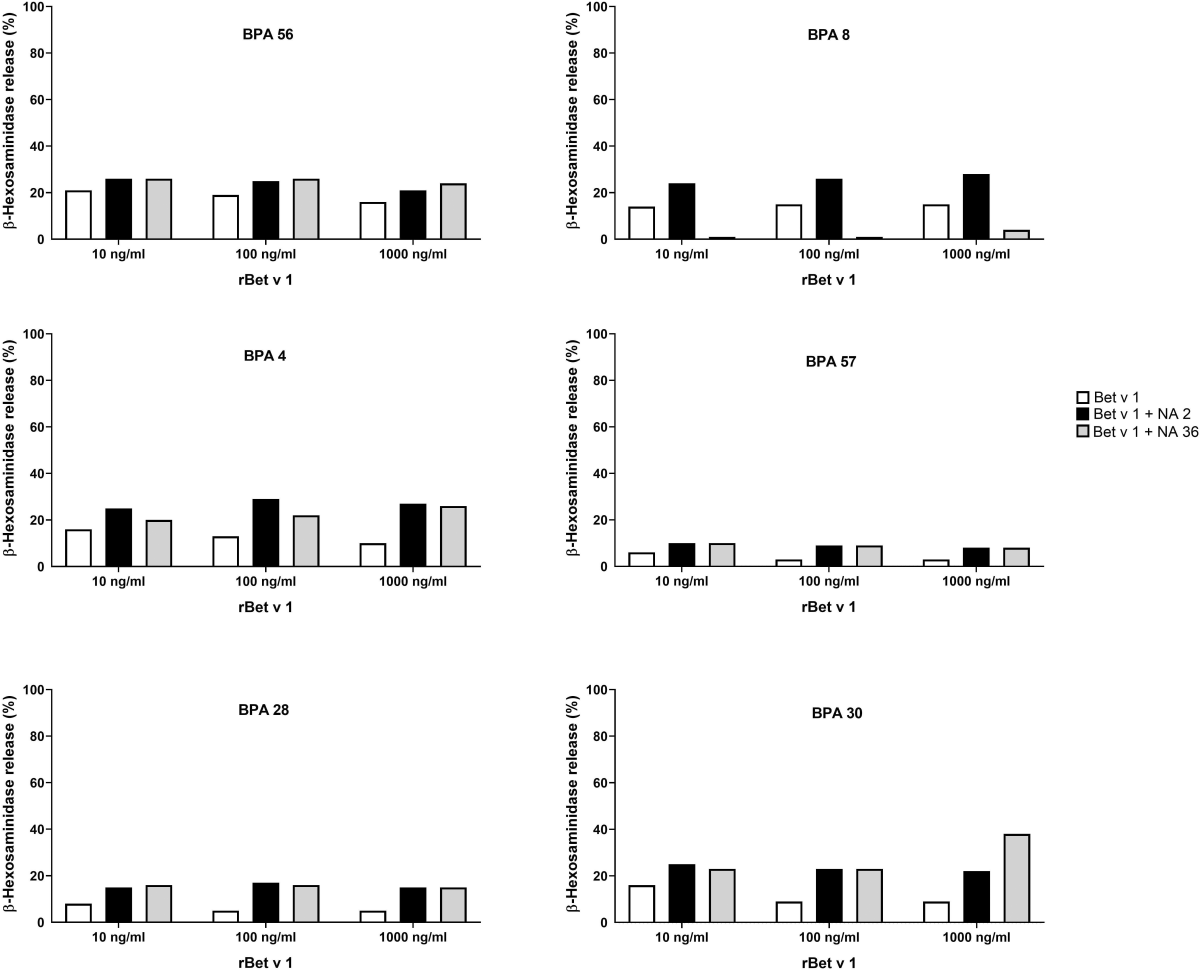
Effects of serum antibodies from non‐allergic subjects on Bet v 1‐induced basophil activation in BPA patients. Shown are percentages of ß‐hexosaminidase release (y‐axes) obtained in basophils which had been loaded with serum IgE from BPA patients (top of Figure) and which were subsequently challenged with Bet v 1 (white bars) or with Bet v 1 which had been pre‐incubated with sera from non‐allergic subjects (grey and black bars) (x‐axes: different concentrations).

Next, we studied the effects of serum antibodies from non‐allergic subjects on Bet v 1‐induced basophil activation in BPA patients. For this purpose, we used a model based on rat basophils expressing human FcεRI which can be loaded with serum IgE from allergic patients and exposed the cells which had been loaded with Bet v 1‐specific IgE with Bet v 1 and Bet v 1 in the presence of serum from non‐allergic subjects as described.^35^, ^36^, ^37^ Results obtained for different concentrations of Bet v 1 and 6 BPA patients are shown in Figure 6. Interestingly, we observed for the majority of tested sera from non‐allergic subjects an enhancement of Bet v 1‐induced basophil activation for each of the three tested Bet v 1 concentrations (Figure 6). Only serum from one non‐allergic subject (i.e. NA36) inhibited Bet v 1‐induced basophil degranulation in allergic patient BPA8 almost completely whereas this serum enhanced degranulation in the other 5 BPA patients (Figure 6) also suggesting considerable variability of the blocking capacity of sera from non‐allergic subjects on Bet v 1‐specific basophil activation in different allergic patients. Sera from the non‐allergic subjects without addition of Bet v 1 had no effect on Bet v 1‐induced basophil activation (Figure 6).

### 3.10 Serum IgG from Bet v 1‐allergic patients can inhibit to some extent Bet v 1‐induced basophil activation

**FIGURE 7:**
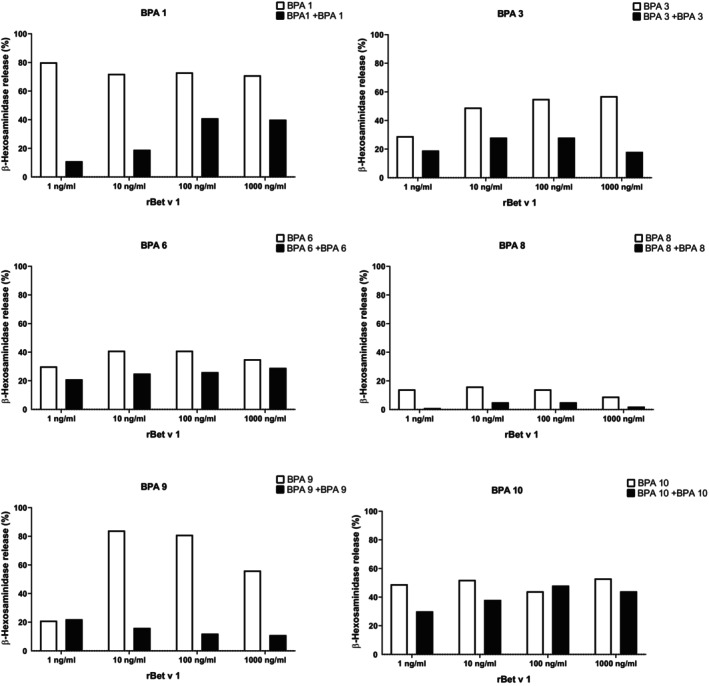
Effects of pre‐incubation of different Bet v 1 concentrations (x‐axes) with IgE‐inactivated sera from Bet v 1‐allergic patients on basophil degranulation (y‐axes: percentages of ß‐hexosaminidase of total release). White bars (Bet v 1 without serum) and black bars (Bet v 1 with heat‐inactivated serum) (averages of duplicates with variations of <10%) show the percentages of mediator release induced (y‐axes).

In a next set of experiments, we studied the effects of allergic patients natural Bet v 1‐specific IgG on Bet v 1‐induced basophil activation in the very same allergic patients. We found that natural Bet v 1‐specific IgG of allergic patients can inhibit to some extent IgE‐mediated basophil activation by Bet v 1 (Figure 7). For BPA 9, basophil activation at 1 ng/mL of Bet v 1 was slightly higher when Bet v 1‐was pre‐incubated with IgE‐heat‐inactivated serum (Figure 7, black bar) as compared to addition of allergen alone (Figure 7, white bar).

## 4 DISCUSSION

The nature of epitopes on the major birch pollen allergen Bet v 1 recognized by natural IgG antibodies of birch pollen allergic patients and birch pollen‐exposed but non‐sensitized subjects has not been studied in detail. The goal of our study was to investigate IgE and IgG recognition of Bet v 1 and to study the effects of natural Bet v 1‐specific IgG antibodies on IgE recognition of Bet v 1 and Bet v 1‐induced basophil activation. To the best of our knowledge, this is the first study, which not only compares allergen‐specific IgE and IgG epitopes recognized by sensitized allergic patients, allergic not‐Bet v 1‐sensitized and non‐allergic subjects but also studied the effects of naturally occurring allergen‐specific IgG antibodies on allergen‐specific IgE binding and allergen‐induced basophil activation in allergic patients.

Our study reveals that IgE antibodies from BPA patients react almost exclusively with conformational epitopes on folded Bet v 1 but not with unfolded Bet v 1 fragments or Bet v 1‐derived synthetic peptides whereas IgG, IgG_1_ and also IgG_4_ antibodies from NBPA and NA subjects show primarily reactivity to unfolded Bet v 1 fragments and peptides as also evidenced by higher cumulative reactivity to unfolded antigens as compared to folded Bet v 1. It should be noted at this place that it is a limitation of our study that we have analysed not all IgG subclass reactivity and IgA but IgG_1_ and IgG_4_ are very important in allergy although they have different functions.^38^ IgG inhibition studies document that IgG binding to folded Bet v 1 is inhibited best by folded Bet v 1 but not by the mix of unfolded F1 and F2 whereas IgG binding to the mix of unfolded F1 and F2 is best inhibited by F1 + F2 and not by folded Bet v 1 (Figure S3). Pre‐incubation of sera with Bet v 1 even seemed to enhance the IgE binding to the fragments probably due to formation of immune complexes (Figure S3). Results from the IgG inhibition studies are interesting because they suggest that IgG reactivity to unfolded Bet v 1 fragments may in part be directed to cryptic epitopes which become available only after fragmentation or denaturation of the allergen whereas the IgG epitopes recognized on folded Bet v 1 are exposed on the surface of the folded allergen. Thus, Bet v 1‐specific IgG antibodies are directed mainly to non‐conformational but also to conformational epitopes. IgG epitopes on folded Bet v 1 do not seem to overlap much with IgE epitopes because natural IgG antibodies could not completely block IgE‐mediated basophil degranulation (Figure 7). These findings are in agreement with results obtained in a study investigating IgE and IgG recognition of major house dust mite allergens, which also demonstrated that IgE reacts with conformational epitopes on intact and folded HDM allergens whereas IgG reacted also with sequential peptide epitopes.^10^ However, HDM specific IgG levels and thus HDM peptide‐specific IgG are usually higher than Bet v 1‐ and Bet v 1‐peptide‐specific IgG levels in our study. Furthermore, the latter HDM study has not investigated IgG subclass reactivity and hence could not inform about possible differences regarding IgG_1_ antibodies, which do not have the same Th2‐dependency as IgG_4_ antibodies.^39^ Nevertheless, we think that our results may be extrapolated in particular to respiratory allergen sources and allergen molecules where patients IgE antibodies mainly recognize conformational epitopes.

We found that the B‐cell epitopes of Bet v 1 determined for IgE (i.e. conformational) and IgG (i.e. non‐conformational and sequential) differ in allergic and non‐sensitized subjects. Accordingly, they obviously must be recognized by different variable regions of the corresponding antibodies. One must therefore conclude that IgE and IgG as well as IgG_1_ and IgG_4_ producing Bet v 1‐specific B cells must have different clonal origin and eventually have evolved from different IgM precursor cells supporting the concept that class‐switching in allergic patients, unlike in sensitized mice, occurs via a non‐sequential pathway.^39^, ^40^, ^41^ The fact that there was some low correlation between Bet v 1‐specific IgE and Bet v 1‐specific IgG_4_ but not for Bet v 1‐specific IgE and IgG_1_ may be due to the fact that class switch to IgE and IgG_4_ may be driven by similar pathways.^42^

How can it be explained that allergen‐specific IgE and IgG antibodies recognize different epitopes on Bet v 1? One possibility could be that Bet v 1‐specific IgG is induced by food containing Bet v 1‐related allergens such as Mal d 1 from apple. However, there was no correlation between IgG reactivity to Bet v 1 and Mal d 1 peptides or Bet v 1 peptide‐specific IgG was higher than Mal d 1 peptide‐specific IgG (Figure S5) which indicates that the induction of IgG specific for Bet v 1 does not occur by Mal d 1 via the gastrointestinal tract. Another possibility is that the immune system encounters larger quantities of unfolded/degraded allergen which is known to induce preferentially IgG responses^43^, ^44^ due to disturbed epithelial barrier.^4^

Another important result of our study is that BPA patients had significantly higher Bet v 1‐specific IgG, IgG_1_ and IgG_4_ antibody levels than NBPA and NA subjects, which confirms results obtained for different allergen molecules from different allergen sources by several other studies.^18^, ^45^, ^46^, ^47^ Thus, taken our results and those of others together there is strong support for the hypothesis that patients who are IgE‐sensitized to a particular allergen also show a more pronounced IgG response to the very same allergen. This is best explained by the fact that allergen‐specific immune responses in allergic patients are regulated by allergen‐specific genetic factors, in particular by MHC antigens responsible for allergen presentation, which has been postulated already long time ago in population‐based genetic association studies^48^, ^49^ and recently got considerable support by experimental data obtained in an allergen‐specific humanized mouse model.^50^

Another important and novel aspect of our work was to study the effects of natural allergen‐specific IgG antibodies on the binding of allergen‐specific IgE antibodies to the corresponding allergen and allergen‐specific and IgE dependent cellular activation. When we investigated the ability of natural Bet v 1‐specific IgG antibodies from NA subjects regarding their ability to inhibit IgE binding of BPA patients to Bet v 1, we found that natural IgG only poorly inhibited the IgE binding of BPA patients to Bet v 1. Only for few NA subjects and certain BPA patients an IgE inhibition of more than 40% was observed and the inhibition of IgE binding varied depending on what BPA‐ and NA subject was tested. Importantly, inhibition of IgE binding did not depend on the titers of allergen‐specific IgG and hence seemed to depend rather on epitope specificity and/or avidity of antibodies. Moreover, we observed for certain BPA and NA subjects that antibodies from NA subjects could even enhance IgE binding of BPA patients to Bet v 1. The results obtained in the molecular interaction assays (i.e. ELISA) were confirmed by cellular assays using basophils, which had been loaded with IgE from BPA patients. In these experiments, we also found that Bet v 1‐induced basophil activation was only rarely inhibited by serum antibodies from non‐allergic subjects and even enhancement of allergen‐specific basophil activation by serum antibodies was observed. Similar results were obtained when the effect of IgG‐containing serum from Bet v 1‐allergic patients in which IgE had been heat‐inactivated was studied on basophil degranulation in the very same patient. Also in allergic patients, IgG‐containing serum reduced to some extent Bet v 1‐induced basophil activation but could not fully suppress it (Figure 7).

At least two possibilities for the enhancement of Bet v 1‐induced basophil activation by serum antibodies come into mind: One possibility is that allergen‐specific IgG antibodies binding to different epitopes on the allergen as compared to IgE may induce a super‐crosslinking as has been demonstrated in a defined cellular experimental model^51^ and was suggested as a possible mechanism for immunoregulation of allergen‐specific IgE responses by allergen‐specific IgG.^41^ The second, non‐mutually exclusive possibility is that allergen‐specific IgG upon binding to Bet v 1 may induce a conformational change in the allergen leading to exposure of additional IgE epitopes as has been demonstrated for Bet v 1‐specific monoclonal antibodies obtained from mice and AIT‐treated patients.^52^, ^53^, ^54^ The demonstration that allergen‐specific IgG antibodies can regulate allergen‐specific IgE‐mediated cellular immune responses seems to us very important because as demonstrated in our study regarding allergen‐specific effector cell responses, it may also contribute to the activation of IgE‐producing memory B cells by super‐cross‐linking of their B‐cell receptors.^41^

Regulation of allergen‐specific IgE‐mediated effector cell responses or failure of blocking of allergen‐specific IgE‐induced mast cell activation by natural IgG is important regarding several aspects. First, it may explain the lack of blocking the IgE allergen interaction by natural IgG in allergic patients, which explains why natural IgG is not or insufficiently protecting against allergy. In this context, we would like to remind of a classical experiment showing that transfer of IgE from ragweed pollen allergic patients to non‐allergic subjects induced allergen‐specific skin sensitivity, which could be only inhibited with serum from AIT‐treated persons.^55^ In this context, we recently reported that vaccination of non‐allergic subjects whose natural IgG antibodies were not protective with hypoallergenic recombinant Bet v 1 fragments could induce IgG responses blocking allergic patients´ IgE binding to Bet v 1 and Bet v 1‐induced basophil activation.^36^ The success of AIT may therefore at least in part depend on the induction of allergen‐specific IgG antibodies which effectively can block IgE binding to the allergen which eventually was not successfully achieved with all birch pollen‐specific AIT vaccines and also not for all Bet v 1‐cross‐reactive PR10 allergens.^16^, ^17^ Second, our study demonstrates that certain non‐allergic subjects, although rare, can be identified by IgE ELISA competition experiments, which contain natural antibodies capable of inhibiting allergen‐induced effector activation. Such protective antibodies may be induced/enhanced by AIT vaccination and used for obtaining immunoglobulin preparations protecting against allergy similar as has been reported for recombinant therapeutic human monoclonal IgG antibodies by passive immunization.^19^, ^20^

It is a limitation of our study that the study population shown in Table 1 comprised a rather wide range of ages but comparable results were obtained in a second study population (Table S2), and it is therefore unlikely that age, sex or origin of the study population had effects on the key findings of our study.

In summary, our study has revealed novel results regarding the natural allergen‐specific IgG responses in allergic and non‐allergic subjects, which have also important implications for allergen‐specific immunotherapy by active and passive immunization.

## AUTHOR CONTRIBUTIONS

RV, GB, MK and RC designed the research studies. GB, AL, ES, AS, AP, YZ, RC, MF‐T, OA, MW, OE, SA, IS, NS, VS, EF, TSL, ECB, VVN, TJ, IT and MT performed clinical and/or experimental work. RV, RC, GB, MK and AK analysed and interpreted the data. GB wrote the manuscript with contributions from RC and RV. All authors read the article and approved the submitted version.

## ACKNOWLEDGEMENTS

None.

## FUNDING INFORMATION

This study was supported by the FWF‐funded project P34472‐B from the Austrian Science Foundation (FWF), by the Danube ARC grant of the country of Lower Austria and by the Megagrant of the Government of the Russian Federation, grant number 075‐15‐2021‐632 (14.W03.31.0024) and, regarding the micro‐array analyses, by a grant from the Russian Science Foundation (Project: No: 23‐75‐30016: ‘Allergen micro‐array‐based assessment of allergic sensitization profiles in the Russian Federation as basis for personalized treatment and prevention of allergy (AllergochipRUS)’).

## CONFLICT OF INTEREST STATEMENT

Rudolf Valenta has received research grants from Viravaxx AG, Vienna, Austria, HVD Biotech, Vienna, Austria and Worg Pharmaceuticals, Hangzhou, China. He serves as a consultant for Viravaxx and Worg. The other authors have not conflicts of interest to declare. The authors with Russian affiliation declare that they have prepared the article in their ‘personal capacity’ and/or that they are employed at an academic/research institution where research or education is the primary function of the entity.

## Supporting information


Figure S1.

Table S1.

## Data Availability

The data that support the findings of this study are available from the corresponding author upon reasonable request.
