# Supplementary figures and images for: Natural human Bet v 1‐specific IgG antibodies recognize non‐conformational epitopes whereas IgE reacts with conformational epitopes
Source: Allergy. 2023 Sep 13;78(12):3136–53. doi: 10.1111/all.15865 (PMC10952721; doi:10.1111/all.15865)

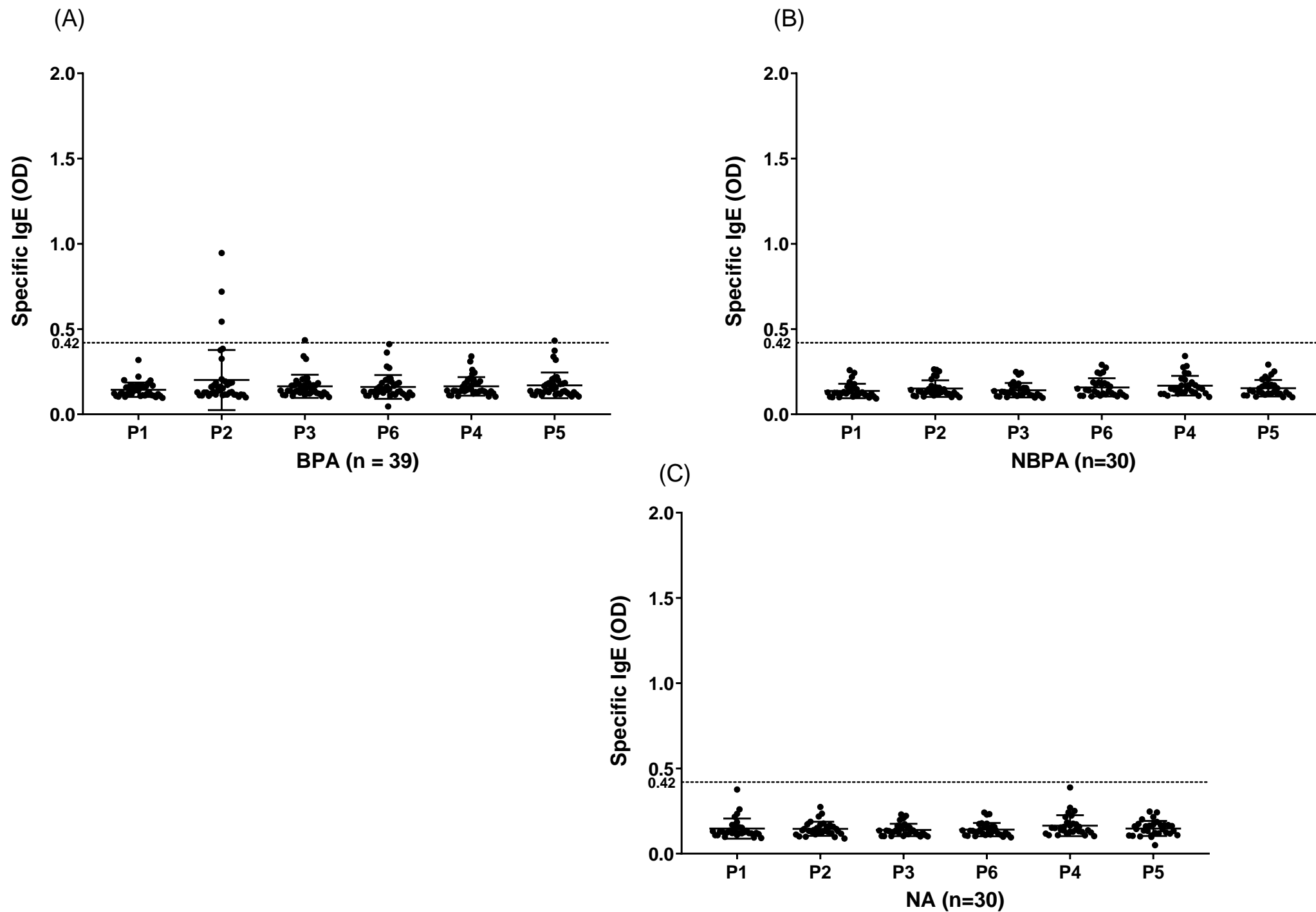

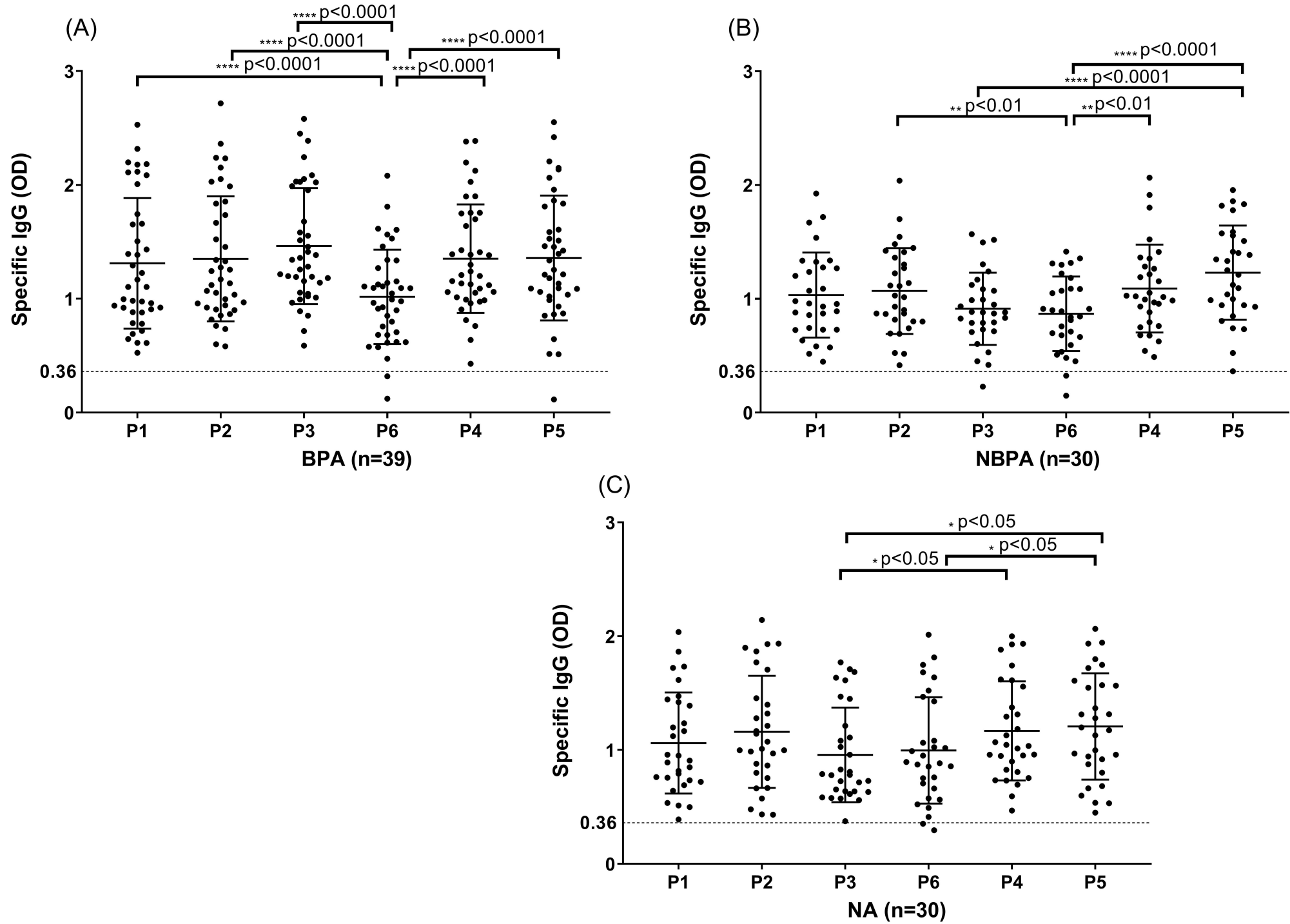

Figure S3

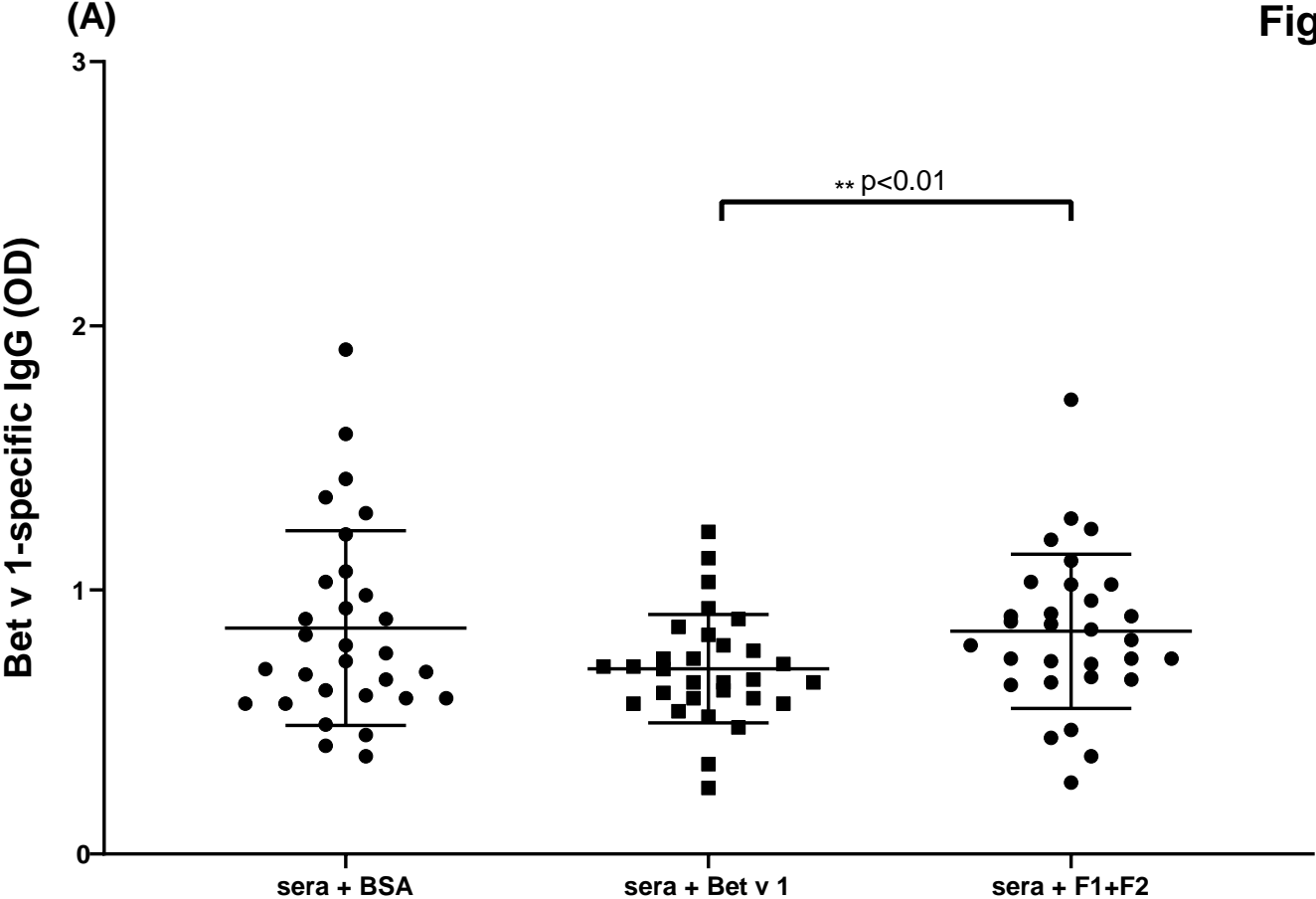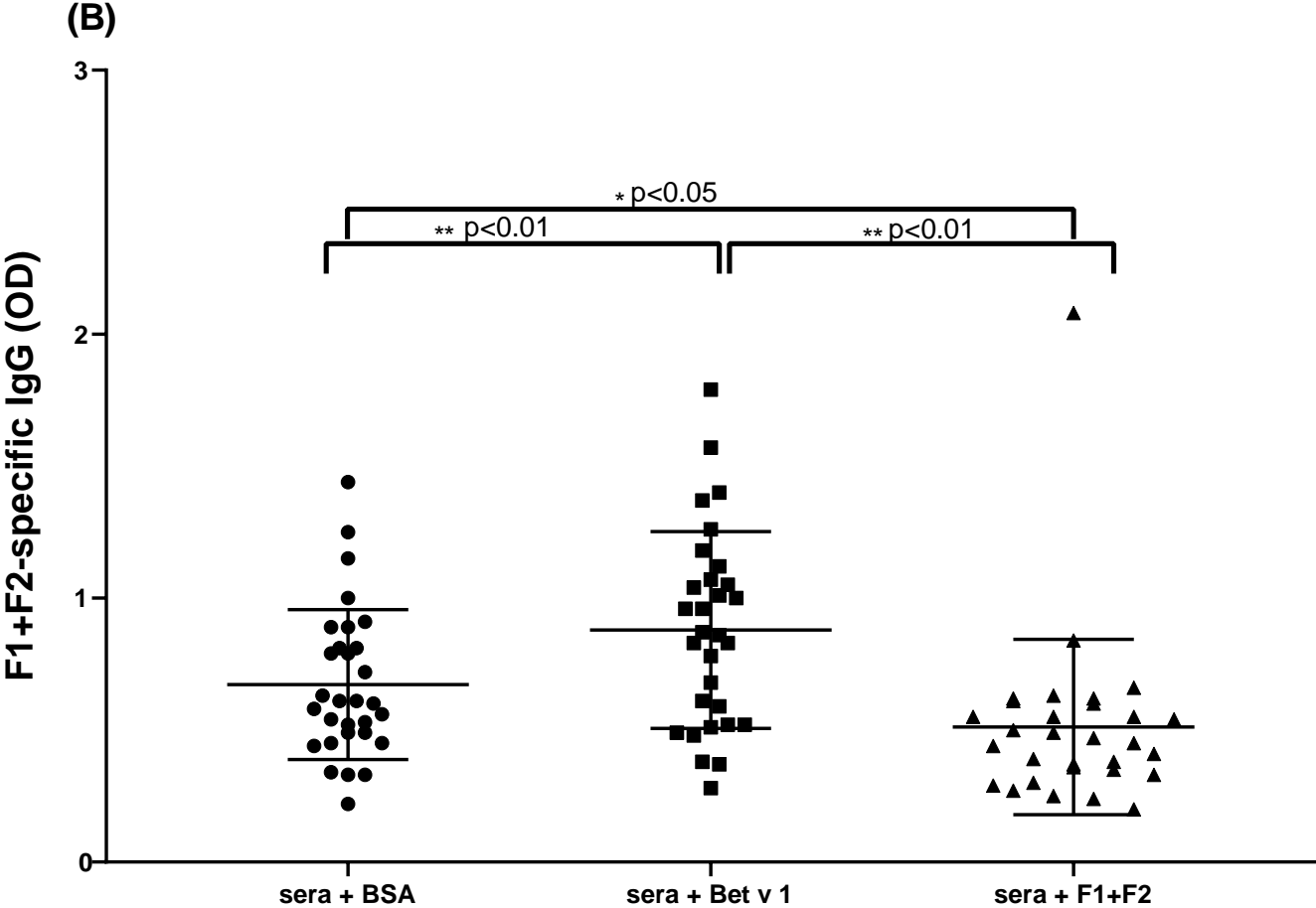

**Figure S4**

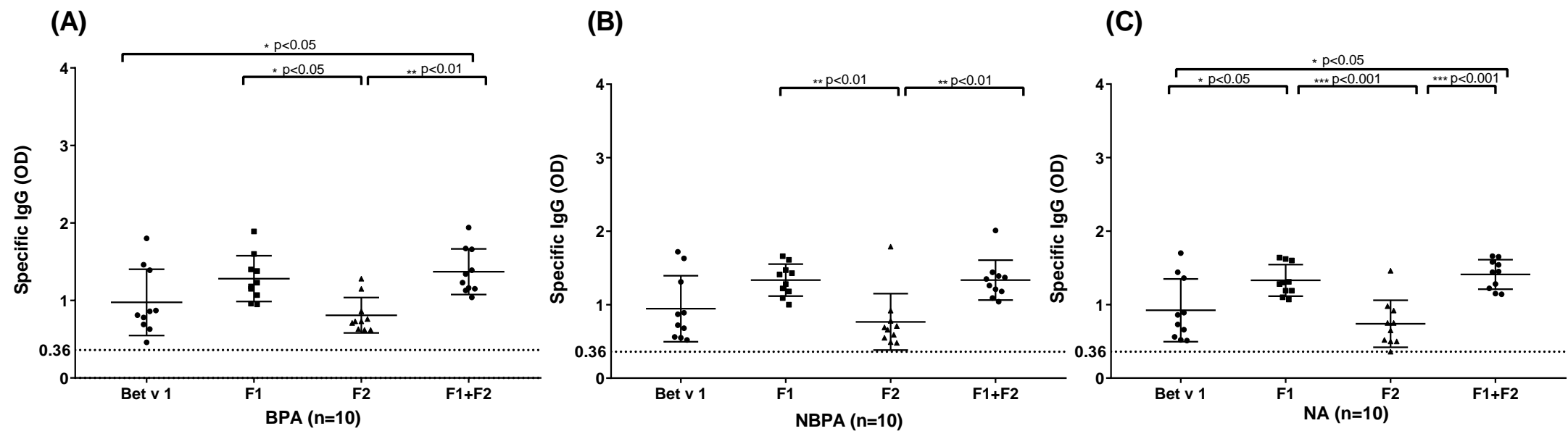

Figure S5

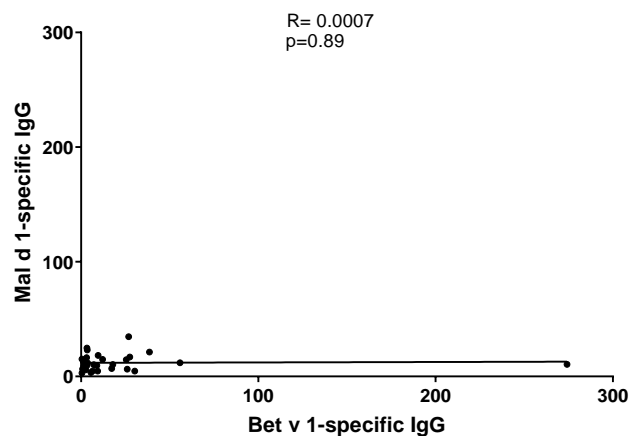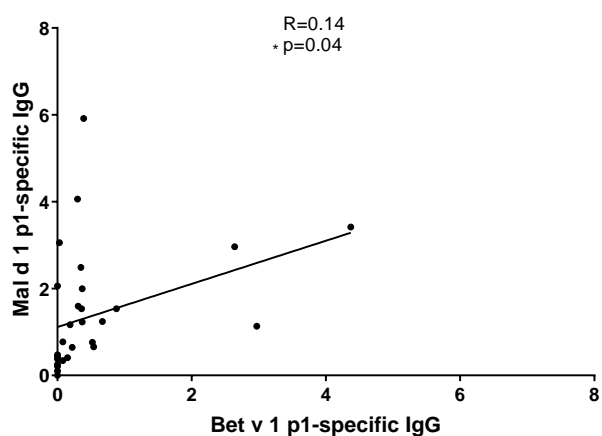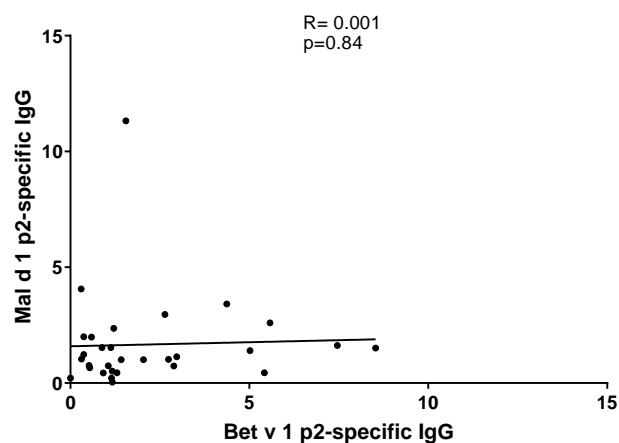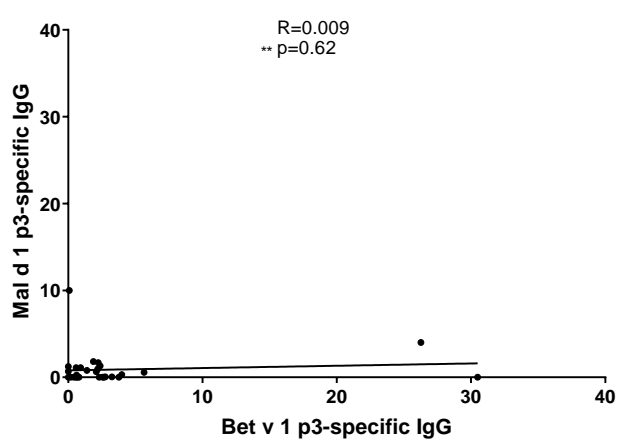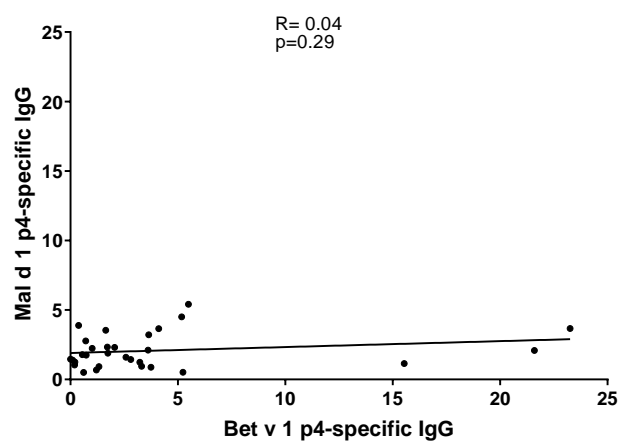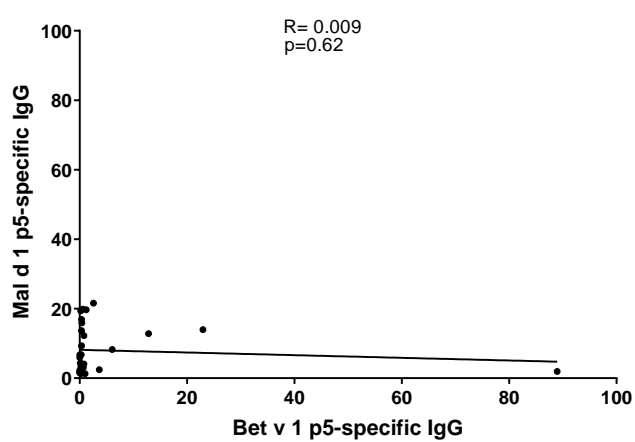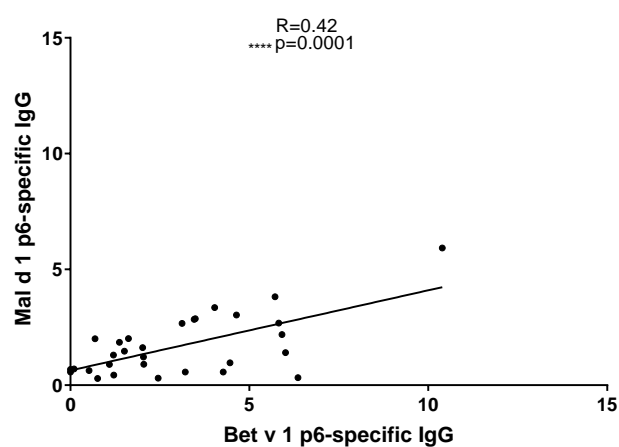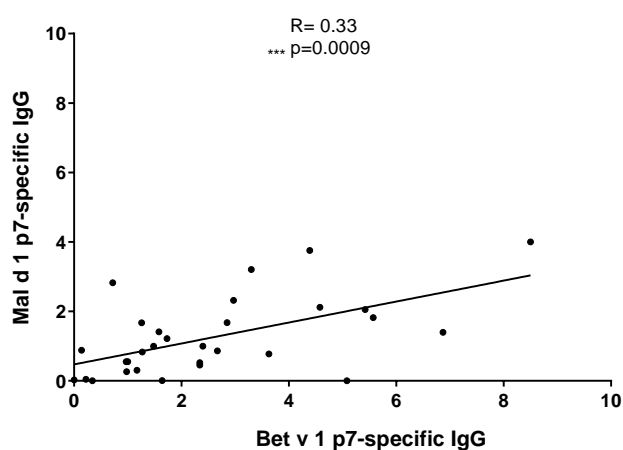

Supplement: Supplementary file 1 — Figure S1. [file ALL-78-3136-s002.pdf]
