# Supplementary material for: Natural human Bet v 1‐specific IgG antibodies recognize non‐conformational epitopes whereas IgE reacts with conformational epitopes
Source: Allergy. 2023 Sep 13;78(12):3136–53. doi: 10.1111/all.15865 (PMC10952721; doi:10.1111/all.15865)
Supplement: Supplementary file 2 — Table S1. [file ALL-78-3136-s001.pdf]

Table S1A. Detailed demographic, clinical and serological characteristics of BPA patients

Patient	Gender	Age	Total IgE (IU/ml)	sIgE to Bet v 1 ISU	Symptoms					Sensitizations					
					Asthma	Rhinoconjunctivitis	Dermatitis	Food Allergy	OAS	Trees	Grass	Weeds	HDM	Dog	Cat
1	m	10	1120	136,63	-	+	+	-	+	+	+	+	+	-	-
2	f	15	13000	144,29	-	+	+	+	+	+	+	+	+	+	+
3	m	14	170	147,66	+	+	-	-	+	+	-	-	+	+	+
4	f	11	590	129	+	+	-	-	-	+	+	-	-	+	+
5	f	12	1000	153,46	-	+	+	-	+	+	+	+	-	+	+
6	m	14	303	169,19	-	+	-	-	+	+	+	+	-	+	+
7	f	15	35	1,04	-	+	-	-	-	+	+	-	-	-	-
8	m	16	3200	111,18	+	+	+	-	-	+	-	-	+	+	-
9	f	15	280	78,22	-	+	-	-	+	+	+	+	+	+	+
10	f	10	231	76,19	-	+	-	-	-	+	+	+	-	-	+
11	m	13	542	53,69	+	+	-	-	-	+	+	+	+	-	+
12	m	14	615	55,1	+	+	+	-	+	+	+	-	+	+	-
13	m	15	10000	57,2	+	-	+	+	+	+	+	+	+	+	+
14	m	10	200	139,89	-	+	-	-	+	+	+	-	-	-	+
15	f	16	650	59,86	-	+	-	-	+	+	-	+	+	-	+
16	m	10	2000	145,71	+	+	+	+	+	+	+	+	+	+	+
17	m	10	50	37,89	+	+	-	-	-	+	+	-	+	-	-
18	m	17	150	14,3	-	+	-	-	+	+	-	-	+	-	-
19	f	10	95	44,93	-	+	-	-	-	+	-	-	-	-	-
20	m	14	930	52,47	-	+	-	-	+	+	+	-	+	+	+
21	f	14	136	37,18	-	+	+	-	+	+	-	-	+	-	+
22	m	12	130	35,09	+	+	-	-	+	+	+	+	+	+	+
23	m	10	980	37,71	-	+	+	-	+	+	+	+	-	+	+
24	m	10	110	40,58	+	+	+	-	-	+	-	-	+	+	+
25	f	10	208	51,01	+	+	-	-	-	+	-	+	-	-	-
26	f	11	200	148,06	-	+	-	-	+	+	-	+	+	+	+
27	m	10	60	41,54	+	+	-	-	-	+	-	+	-	-	-
28	f	15	78	5,55	+	+	-	-	-	+	-	-	-	-	+
29	m	17	133	6,32	-	+	-	-	-	+	-	-	-	+	-
30	m	16	124	3,43	-	+	-	-	-	+	-	-	+	-	+
31	f	9	44,4	2,29	+	+	+	-	-	+	+	-	-	-	-
32	m	14	50	37,2	-	+	-	-	+	+	-	-	-	-	-
33	m	8	127,3	171	+	+	+	+	+	+	-	+	-	-	-
34	f	7	109,5	100	-	+	-	-	+	+	-	+	-	+	+
35	m	5	62,3	50,6	+	+	+	+	+	+	-	-	-	+	+
36	f	5	63,3	100	-	+	-	-	+	+	-	-	-	+	+
37	m	6	103,2	100	+	+	+	+	+	+	-	-	-	-	-
38	m	5	553	30	-	+	+	+	+	+	+	-	-	-	+
39	f	5	18,7	2,83	-	+	+	-	-	+	-	-	-	-	-
40	m	35	900	NA	-	+	+	-	-	+	-	-	+	+	+
41	m	26	4400	85,96	+	+	+	+	+	+	+	+	+	+	+
44	f	21	NA	NA	+	+	+	-	+	+	+	+	+	+	+
45	m	10	126	NA	+	+	+	-	+	+	+	-	-	-	-
46	m	44	287	3,28	-	+	+	-	+	+	+	+	-	-	-
47	m	34	1939	NA	-	+	-	+	+	+	-	-	-	-	-
48	m	33	NA	NA	+	+	+	-	-	+	+	+	+	+	+
49	m	12	10100	54	+	+	+	-	+	+	-	-	+	-	+
50	m	38	NA	NA	+	+	+	+	-	+	+	+	+	-	+
51	m	35	NA	41,21	+	+	-	-	+	+	+	-	-	-	-
52	f	19	NA	NA	-	+	+	-	+	+	-	-	-	-	-
53	f	22	134	98,1	-	+	-	-	+	+	+	+	-	-	+
54	f	55	NA	19,18	-	+	+	-	+	+	-	-	-	-	-
55	f	10	121	1,5	-	+	+	+	+	+	-	-	-	-	-
56	m	13	341	NA	-	+	+	-	-	+	-	-	+	-	-
57	m	45	NA	35,3	-	+	+	-	+	+	+	+	+	+	+
58	m	19	191	NA	+	+	+	-	-	+	-	-	+	+	+
59	f	29	164	NA	-	+	+	+	+	+	+	-	+	-	-
60	f	22	NA	35,9	-	+	+	-	-	+	+	-	-	-	-
61	f	30	123	2,01	-	+	+	-	-	+	-	-	-	-	+
62	f	36	NA	NA	-	-	+	-	-	+	+	-	+	+	+
63	m	33	NA	0,48	-	+	+	-	-	+	-	-	+	-	+
64	m	13	NA	5,9	+	+	-	-	+	+	-	-	+	+	+
65	m	15	420	26,02	+	+	+	-	+	+	+	-	-	-	-
66	f	17	1255	64,06	+	+	+	+	+	+	+	+	-	+	+

m: Male, f: Female, OAS: Oral allergy syndrome, HDM: House dust mite, NA: Not available

Table S1B. Detailed demographic, clinical and serological characteristics of NBPA patients

Patient	Gender	Age	Total IgE (IU/ml)	Symptoms					Sensitizations					
				Asthma	Rhinoconjunctivitis	Dermatitis	OAS	Food Allergy	Trees	Grass	Weeds	HDM	Dog	Cat
1	m	16	75	+	+	-	-	-	-	-	-	+	-	-
2	m	15	201	-	-	+	-	-	-	+	-	+	+	+
3	f	10	60	-	-	+	+	-	-	-	-	-	-	+
4	f	11	100	-	+	+	-	+	-	-	-	+	+	+
5	m	11	120	+	+	+	-	-	-	+	+	+	+	+
6	m	14	81	+	+	-	-	-	-	-	-	+	-	+
7	m	10	22	+	+	+	-	-	-	-	-	+	-	-
8	f	10	138	-	+	+	-	-	-	+	-	-	-	+
9	f	10	35	-	+	+	-	-	-	-	-	-	-	+
10	m	15	62	-	+	+	-	-	-	-	-	-	-	-
11	m	11	89	+	-	+	-	-	-	-	-	+	-	-
12	m	11	277	-	+	-	-	-	-	-	-	+	-	+
13	f	11	24	-	+	-	-	-	-	-	-	-	+	+
14	m	11	52	+	+	-	-	-	-	-	+	+	+	+
15	f	13	36	-	+	+	-	-	-	+	-	-	+	+
16	f	15	22	+	+	-	-	-	-	-	-	+	-	-
17	f	16	55	-	+	-	-	-	-	-	-	-	-	+
18	m	16	32	-	+	-	-	+	-	-	-	-	-	-
19	f	13	32	-	-	-	-	+	-	-	-	-	-	-
20	f	16	30	-	+	-	-	-	-	-	-	+	-	-
21	m	25	NA	-	+	-	-	-	-	-	-	+	-	-
22	f	14	200	+	+	-	-	-	-	-	+	+	+	-
23	m	10	25	+	+	-	-	-	-	-	+	-	-	-
24	f	11	1000	-	+	+	-	-	-	+	-	-	-	+
25	m	16	100	-	+	-	-	-	-	+	+	-	-	-
26	f	10	25	-	+	-	-	-	-	-	-	+	-	-
27	m	13	6	-	+	-	-	-	-	+	-	-	-	-
28	m	10	3.5	+	+	-	-	-	-	-	-	-	-	-
29	f	14	140	+	+	+	-	-	-	-	-	-	-	+
30	m	30	322	+	+	+	-	-	-	-	+	+	+	+

IgE reactivity (ISU-IgE) (cut off: 0.3)

- Trees
- Grass
- Weeds
- HDM
- Dog
- Cat

m: Male
f: Female
OAS: Oral allergy syndrome
HDM: House dust mite
NA: Not available

Patient	Allergen-specific IgE (ISU)																
	Bet v 1	Act d 1	Act d 2	Alt a 1	Alt a 6	Amb a 1	V nAna o 2	r Ana o 3	Api g 1	Asp f 6	Bla g 5	Blo t 5	Bos d 6	Can f 1	Can f 2	Can f 3	V Can f 4
1	0	0	0	0	0	0	0	0	0	0	0	0	0	0	0	0	0
2	0	0	0	0	0	0	0	0	0	0	0	0	0	2.41	0	0	2.1
3	0	0	0	0	0	0	0.7	1.81	0	0	0	0	0	0	0	0	0
4	0	0	0	0	0	0	0	0	0	0	0	0	0	0	0	0	0
5	0.15	0	0	0	0	0.21	0	0	0	0	0	1.93	0.62	35.37	0.35	11.02	0.18
6	0	0	0	0	0	0	0	0	0	0	0	5.49	0	0	0	0	0
7	0	0	0	0	0	0	0	0	0	0	0	0	0	0	0	0	0
8	0	0	0	0	0	0	0	0	0	0	0	0	0	0	0	0	0
9	0	0	0	0	0	0	0	0	0	0	0	0	0	0	0	0	0
10	0	0	0	0	0	0	0	0	0	0.64	0	0	0	0	0	0	0
11	0	0	0	0	0	0	0	0	0	0	0	4.81	0	0	0	0	0
12	0	0	0	0	0	0	0	0	0	0	0	0	0	0	0	0	0
13	0	0	0	0	0	0	0	0	0	0	0	0	0	0	0	0	0
14	0	0	0	0	0	0	0	0	0	0	0	0	0	1.87	0	0	0
15	0	0	0	0	0	0	0	0	0	0	0	0	0	1.33	0	0	0
16	0	0	0	0	0	0	0	0	0	0	6.59	0	0	0	0	0	0
17	0	0	0	0	0	0	0	0	0	1.93	0	0	0	0	0	0	0
18	0	0	0	0	1.25	0	0	0	0	0	0	0	0	0	0	0	0
19	0	0	0	0	0	0	0	0	0	0	0	0	0	0	0	0	0
20	0	0	0	0	0	0	0	0	0	0	0	0	0	0	0	0	0
21	NA	NA	NA	NA	NA	NA	NA	NA	NA	NA	NA	NA	NA	NA	NA	NA	NA
22	NA	NA	NA	NA	NA	NA	NA	NA	NA	NA	NA	NA	NA	NA	NA	NA	NA
23	0	0	1.88	17.27	0.63	3.07	0	0	0	0	0	0	0	0	0	0	0.21
24	0	0	0.16	0	0	0	0	0	0	0	0	0	0	0	0	0	0
25	NA	NA	NA	NA	NA	NA	NA	NA	NA	NA	NA	NA	NA	NA	NA	NA	NA
26	0	0	0	0	0	0	0	0	0	0	0	0	0	0	0	0	0
27	0	0	0	0	0	0	0	0	0	0	0	0.18	0	0	0	0	0
28	0	0.83	0	0	0	0	0	0	1.22	0	0	0	0	0	0	0	0
29	0	0	0	0	0	0	0	0	0	0	0	0	0	0	0	0	0
30	NA	NA	NA	NA	NA	NA	NA	NA	NA	NA	NA	NA	NA	NA	NA	NA	NA

Patient	Can f 5	Cor a 9	Cor a 14	Cyn d 1	Der f 1	Der f 2	Der p 1	V rAna o 2	Der p 2	V Der p 5	V Der p 7	V Der p 21	V Der p 23	V clone 16
1	0	0	0	0	3.07	4.31	2.12	0	4.48	0.94	0.83	0	0	0
2	1.4	0	0	0.47	14.78	16.24	12.51	0.77	15.43	5.55	2.67	0	4.66	0.44
3	0	0.83	4.21	0	0	0	0	0	0	0	0	0	0	0
4	2.36	0	0	0	0	0	0	0	0	0	0	0	0	0
5	0	0	0	0	0	0	0	0	0	0	0	0	0	0
6	0	0	0	0	0	1.28	0	0	0.62	4.55	0.98	7.34	0	0
7	0	0	0	0	0	0	0	0	0	0	0	0	0	0
8	0	0	0	0	0	0	0	0	0	0	0	0	0	0
9	0	0	0	0	0	0	0	0	0	0	0	0	0	0
10	0	0	0	0	0	0	0	0	0	0	0	0	0	0
11	0	0	0	0	0	1.27	0	0	0.97	5.04	1	5.26	0	1.02
12	0	0	0	0	0	0	0	0	0	0	0	0	0	0
13	2.33	0	0	0	0	0	0	0	0	0	0	0	0	0
14	0	0	0	0	0	0	0	0	0	0	0	0	0	0
15	0	0	0	0	0	0	0	0	0	0	0	0	0	0
16	0	0	0	0	0	0	0	0	0	0	0	0	0	0
17	0	0	0	0	0	0	0	0	0	0	0	0	0	0
18	0	0	0	0	0	0	0	0	0	0	0	0	0	0
19	0	0	0	0	0	0	0	0	0	0	0	0	0	0
20	0	0	0	0	0	0	0	0	0	0	0	0	0	0
21	NA	NA	NA	NA	NA	NA	NA	NA	NA	NA	NA	NA	NA	NA
22	NA	NA	NA	NA	NA	NA	NA	NA	NA	NA	NA	NA	NA	NA
23	0	0	0	0	0	0	0	0	0	0	0	0	0	0
24	0	0	0	0.15	0	0	0	0	0	0	0	0	0	0
25	NA	NA	NA	NA	NA	NA	NA	NA	NA	NA	NA	NA	NA	NA
26	0	0	0	0	0.49	0	0.41	0	0	0	0	0	0	0
27	0	0	0	0.21	0	0	0	0	0	0	0	0	0	0
28	0	0	0	0	0	0	0	0	0	0	0	0	0	0
29	0	0	0	0	0	0	0	0	0	0	0	0	0	0
30	NA	NA	NA	NA	NA	NA	NA	NA	NA	NA	NA	NA	NA	NA

Patient	Allergen-specific IgE (ISU)															
	Equ c 1	Equ c 3	Fel d 1	Fel d 2	Fel d 4	V Fel d 7	Gad c 1	Gal d 3	Gly m 4	Gly m 5	Jug r 1	Jug r 2	Jug r 3	Mus m 1	Ole e 9	Pen m 2
1	0	0	0	0	0	0	0	0	0	0	0	0	0	0	0	0
2	0	0	31.73	0.69	0	0	0	0	0	0	0	0	0	0	0	0
3	0	0	2.19	0	0	0	0	0	0	0	23.4	3.59	0.47	0.36	0	0
4	0	0	15.26	0	0	0	0	0	0	0	0	0	0	0	0	0
5	9.42	4.39	68.96	13.41	20.35	30.65	1.6	11.97	0	0	0	0.5	0	6.49	0	0
6	0	0	2.01	0	0	0	0	0	2.17	0.21	0	0	0	0	0	0
7	0	0	0	0	0	0	0	0	0	0	0	0	0	0	0	0
8	0	0	0.42	0	0	0	0	0	0	0	0	0	0	0	0	0
9	0	0	0.29	0	0	0	0	0	0	0	0	0	0	0	0	0
10	0.29	0	0	0	0	0	0	0	0	0	0	0	0	0	0	0
11	0	0	0	0	0	0	0	0	0.46	0.17	0	0	0	0	0	0
12	0.55	0	2.01	0	4.34	0	0	0	0	0	0	0	0	0.26	0	0
13	0	0	3.2	0	2.26	0	0	0	0	0	0	0	0	0	0	0
14	0.54	0	19.89	0.2	0.59	0	0	0	0	0	0	0	0	0	0	0
15	8	0	8.11	0.56	29.71	0.44	0	0.18	0	0	0	0	0	0.62	0	0
16	0	0	0	0	0	0	0	0	0	0	0	0	0	0	0	0
17	0	0	0	0	0.57	0	0	0	0	0	0	0	0	0	0.36	0
18	0	0	0	0	0	0	0	0	0	0.42	0	0	0	0	0	3.02
19	0	0	0	0	0	0	0	0	0	0	0	0	0	0	0	1.34
20	0	0	0	0	0	0	0	0	0	0	0	0	0	0	0	0
21	NA	NA	NA	NA	NA	NA	NA	NA	NA	NA	NA	NA	NA	NA	NA	NA
22	NA	NA	NA	NA	NA	NA	NA	NA	NA	NA	NA	NA	NA	NA	NA	NA
23	0	0	0	0	0	0	0	0	0	0	0	0	0	0	0	0
24	0.41	0	2.9	0	0	0	0	0	0	0	0	0	0.19	0	0	0
25	NA	NA	NA	NA	NA	NA	NA	NA	NA	NA	NA	NA	NA	NA	NA	NA
26	0	0	0	0	0	0	0	0	0	0	0	0	0	0	0	0
27	0	0	0	0	0	0	0	0	0	0	0	0.19	0	0	0	0
28	0	0	0	0	0	0	0	0	0	0	0	0	0	0	0	0
29	0	0	0.46	0	0	0	0	0	0	0	0	0	0	0	0	0
30	NA	NA	NA	NA	NA	NA	NA	NA	NA	NA	NA	NA	NA	NA	NA	NA

Patient	Phl p 1	Phl p 1 Pichia	Phl p 2	Phl p 4	Phl p 5b	Phl p 6	Phl p 7	Phl p 11	Pol d 5	Tri a aA_TI	Ves v 5
1	0	0	0	0	0	0	0	0	0	0	0
2	3.91	0.91	0	0	0	0	0	0	0	0	0
3	0	0	0	0	0	0	0	0	0	0	0
4	0	0	0	0	0	0	0	0	0	0	0
5	0	0	0	0.35	8.94	1.18	0.6	0	0	0	0
6	0	0	0	0	0	0	0	0	0	0	0
7	0	0	0	0	0	0	0	0	0	0	0
8	0	0	0	0.16	0	0	0	0	0	0	0
9	0	0	0	0	0	0	0	0	0	0	0
10	0	0	0	0	0	0	0	0	0	0	0
11	0	0	0	0	0	0	0	0	0.63	0	5.05
12	0	0	0	0	0	0	0	0	0	0	0
13	0	0	0	0	0	0	0	0	0	0	0
14	0	0	0	0	0	0	0	0	0	0	0
15	0	0	0.66	0	0	0	0	0	0	0	0
16	0	0	0	0	0	0	0	0	0.43	0	0.33
17	0	0	0	0	0	0	0	0	0	0	0
18	0	0	0	0	0	0	0	0	0	0	0.48
19	0	0	0	0	0	0	0	0	0	0	0
20	0	0	0	0	0	0	0	0	0.25	0	0.6
21	NA	NA	NA	NA	NA	NA	NA	NA	NA	NA	NA
22	NA	NA	NA	NA	NA	NA	NA	NA	NA	NA	NA
23	0	0	0	0	0	0	0	0	0	0	0
24	0	0	0	0	0	0	0	0.73	1.16	0	1.11
25	NA	NA	NA	NA	NA	NA	NA	NA	NA	NA	NA
26	0	0	0	0	0	0	0	0	0	0	0
27	0	0	0	0	0	0	0	0	0.24	0.83	0.18
28	0	0	0	0	0	0	0	0	0	0	0
29	0	0	0	0	0	0	0	0	0	0	0
30	NA	NA	NA	NA	NA	NA	NA	NA	NA	NA	NA

Table S1C. Detailed demographic, clinical and serological characteristics of NA subjects

Patient	Gender	Age	Total IgE (IU/ml)	Bet v 1 - Medall (ISU)	Symptoms					Sensitizations					
					Asthma	Rhinoconjunctivitis	Dermatitis	Food allergy	OAS	Trees	Grass	Weeds	HDM	Dog	Cat
1	f	13	30	0	-	-	-	-	-	-	-	-	-	-	-
2	m	16	50	0	-	-	-	-	-	-	-	-	-	-	-
3	m	15	45	0	-	-	-	-	-	-	-	-	-	-	-
4	m	13	13	0	-	-	-	-	-	-	-	-	-	-	-
5	m	11	45	0	-	-	-	-	-	-	-	-	-	-	-
6	m	14	50	0	-	-	-	-	-	-	-	-	-	-	-
7	m	29	NA	NA	-	-	-	-	-	-	-	-	-	-	-
8	f	11	15	0	-	-	-	-	-	-	-	-	-	-	-
9	f	14	45	0	-	-	-	-	-	-	-	-	-	-	-
10	f	11	60	0	-	-	-	-	-	-	-	-	-	-	-
11	f	19	5	0	-	-	-	-	-	-	-	-	-	-	-
12	f	15	80	0	-	-	-	-	-	-	-	-	-	-	-
13	m	14	50	0	-	-	-	-	-	-	-	-	-	-	-
14	m	16	22	0	-	-	-	-	-	-	-	-	-	-	-
15	f	15	15	0	-	-	-	-	-	-	-	-	-	-	-
16	f	29	NA	NA	-	-	-	-	-	-	-	-	-	-	-
17	f	45	NA	NA	-	-	-	-	-	-	-	-	-	-	-
18	f	41	NA	NA	-	-	-	-	-	-	-	-	-	-	-
19	f	37	NA	NA	-	-	-	-	-	-	-	-	-	-	-
20	f	44	NA	NA	-	-	-	-	-	-	-	-	-	-	-
21	f	16	NA	NA	-	-	-	-	-	-	-	-	-	-	-
22	f	17	NA	NA	-	-	-	-	-	-	-	-	-	-	-
23	f	18	NA	NA	-	-	-	-	-	-	-	-	-	-	-
24	f	14	NA	NA	-	-	-	-	-	-	-	-	-	-	-
25	f	25	NA	NA	-	-	-	-	-	-	-	-	-	-	-
26	m	25	NA	NA	-	-	-	-	-	-	-	-	-	-	-
27	m	23	NA	NA	-	-	-	-	-	-	-	-	-	-	-
28	f	34	NA	NA	-	-	-	-	-	-	-	-	-	-	-
29	f	27	NA	NA	-	-	-	-	-	-	-	-	-	-	-
30	m	27	NA	NA	-	-	-	-	-	-	-	-	-	-	-
31	m	30	NA	NA	-	-	-	-	-	-	-	-	-	-	-
32	m	23	NA	NA	-	-	-	-	-	-	-	-	-	-	-
33	f	25	NA	NA	-	-	-	-	-	-	-	-	-	-	-
34	f	25	NA	NA	-	-	-	-	-	-	-	-	-	-	-
35	m	25	NA	NA	-	-	-	-	-	-	-	-	-	-	-
36	m	25	NA	NA	-	-	-	-	-	-	-	-	-	-	-
37	f	21	NA	NA	-	-	-	-	-	-	-	-	-	-	-
38	m	32	NA	NA	-	-	-	-	-	-	-	-	-	-	-

m: Male, f: Female, OAS: Oral allergy syndrome, HDM: House dust mite, NA: Not available

Table S2. Demographic and clinical characterization of additionally analyzed BPA, NBPA and NA subjects.

Characteristics of subjects	Group 1 (BPA, n = 10)	Group 2 (NBPA, n = 10)	Group 3 (NA, n = 10)
**Gender**			
Male, no. (%)	6 (60%)	6 (60%)	2 (20%)
Female, no. (%)	4 (40%)	4 (40%)	8 (80%)
**Age (years)**			
Mean (± SD)	40.0 ± 15.1	33.5 ± 15.9	36.0 ± 8.1
Range	27 - 68	12 - 73	26 - 51
**Allergies, no. (%)**			
Trees	10 (100%)	2 (20%)	0 (0%)
Grass	2 (20%)	6 (60%)	0 (0%)
Weeds	1 (10 %)	0 (0 %)	0 (0%)
HDM	2 (20 %)	5 (50%)	0 (0%)
Dog	3 (30%)	1 (10%)	0 (0%)
Cat	3 (30 %)	3 (30%)	0 (0%)
Food Allergy	2 (20 %)	4 (40%)	0 (0%)
**Symptoms, no. (%)**			
Asthma	1 (10 %)	2 (20%)	0 (0%)
Rhinoconjunctivitis	10 (100%)	10 (100%)	0 (0%)
Dermatitis	1 (10%)	0 (0%)	0 (0%)
OAS	1 (10%)	1 (10%)	0 (0%)

Table S 3. Characteristics of Bet v 1-derived peptides (Modified from Focke *et al.*, ref. 25).

Peptides	Position aa	Sequence	No. of aa	MW (Da)	pI
P1	1 – 24	MGVFNYETETTSVIPAARLFKAFI**C**	25	2809.3	6.28
P2	30 - 59	LFPKVAPQAISSVENIEGNGGPGTIKKISF**C**	31	3202.7	8.63
P3	50 - 79	**C**GPGTIKKISFPEGFPFKYVKDRVDEVDHTN	31	3525	7.00
P6	75 - 104	**C**VDHTNFKYNYSVIEGGPIGDTLEKISN EIK	31	3484.9	4.70
P4	110 - 139	DGGSILKISNKYHTKGDHEVKAEQVKASKE**C**	31	3400.8	8.44
P5	130 - 160	**C**KAEQVKASKEMGETLLRAVESYLLAHSDAYN	32	3556.1	5.49

aa: amino acid, MW: molecular weight, pI: Isoelectric point.

Table S4. IgE levels specific for Bet v 1, Bet v 1 fragments and Bet v 1 peptides

Subject	Specific IgE (OD)								
	Bet v 1	F1	F2	P1	P2	P3	P6	P4	P5
BPA 1	1.47	0.00	0.00	0.00	0.00	0.00	0.00	0.00	0.00
BPA 2	1.49	0.00	0.00	0.00	0.00	0.00	0.00	0.00	0.00
BPA 3	0.93	0.00	0.00	0.00	0.00	0.00	0.00	0.00	0.00
BPA 4	2.53	0.00	0.00	0.00	0.00	0.00	0.00	0.00	0.00
BPA 5	1.59	0.00	0.00	0.00	0.00	0.00	0.00	0.00	0.00
BPA 6	1.45	0.00	0.00	0.00	0.00	0.00	0.00	0.00	0.00
BPA 7	3.43	0.00	0.00	0.00	0.00	0.00	0.00	0.00	0.00
BPA 8	2.74	0.00	0.00	0.00	0.00	0.00	0.00	0.00	0.00
BPA 9	0.47	0.00	0.00	0.00	0.00	0.00	0.00	0.00	0.00
BPA 10	1.50	0.00	0.00	0.00	0.00	0.00	0.00	0.00	0.00
BPA 11	1.74	0.47	0.00	0.00	0.30	0.00	0.00	0.00	0.00
BPA 12	0.41	0.00	0.00	0.00	0.00	0.00	0.00	0.00	0.00
BPA 13	1.72	0.83	0.74	0.00	0.53	0.00	0.00	0.00	0.00
BPA 14	0.67	0.00	0.00	0.00	0.00	0.00	0.00	0.00	0.00
BPA 15	1.34	0.05	0.00	0.00	0.00	0.00	0.00	0.00	0.00
BPA 16	1.92	0.00	0.00	0.00	0.00	0.00	0.00	0.00	0.00
BPA 17	0.24	0.00	0.00	0.00	0.00	0.00	0.00	0.00	0.00
BPA 18	0.08	0.00	0.00	0.00	0.00	0.00	0.00	0.00	0.00
BPA 19	0.52	0.00	0.00	0.00	0.00	0.00	0.00	0.00	0.00
BPA 20	0.40	0.00	0.00	0.00	0.00	0.00	0.00	0.00	0.00
BPA 21	0.24	0.00	0.00	0.00	0.00	0.00	0.00	0.00	0.00
BPA 22	0.81	0.00	0.00	0.00	0.00	0.00	0.00	0.00	0.00
BPA 23	1.72	0.68	0.69	0.00	0.12	0.01	0.00	0.00	0.00
BPA 24	0.70	0.17	0.31	0.00	0.00	0.00	0.00	0.00	0.01
BPA 25	0.00	0.00	0.00	0.00	0.00	0.00	0.00	0.00	0.00
BPA 26	1.46	0.00	0.00	0.00	0.00	0.00	0.00	0.00	0.00
BPA 27	0.23	0.00	0.00	0.00	0.00	0.00	0.00	0.00	0.00
BPA 28	1.80	0.00	0.00	0.00	0.00	0.00	0.00	0.00	0.00
BPA 29	0.05	0.00	0.00	0.00	0.00	0.00	0.00	0.00	0.00
BPA 30	0.31	0.00	0.00	0.00	0.00	0.00	0.00	0.00	0.00
BPA 31	0.33	0.00	0.00	0.00	0.00	0.00	0.00	0.00	0.00
BPA 32	0.74	0.00	0.00	0.00	0.00	0.00	0.00	0.00	0.00
BPA 33	0.07	0.00	0.00	0.00	0.00	0.00	0.00	0.00	0.00
BPA 34	0.04	0.00	0.00	0.00	0.00	0.00	0.00	0.00	0.00
BPA 35	0.00	0.00	0.00	0.00	0.00	0.00	0.00	0.00	0.00
BPA 36	0.32	0.00	0.00	0.00	0.00	0.00	0.00	0.00	0.00
BPA 37	2.20	0.00	0.00	0.00	0.00	0.00	0.00	0.00	0.00
BPA 38	0.65	0.00	0.00	0.00	0.00	0.00	0.00	0.00	0.00
BPA 39	0.44	0.00	0.00	0.00	0.00	0.00	0.00	0.00	0.00

Subject	Specific IgE (OD)								
	Bet v 1	F1	F2	P1	P2	P3	P6	P4	P5
NBPA 1	0.00	0.00	0.00	0.00	0.00	0.00	0.00	0.00	0.00
NBPA 2	0.00	0.00	0.00	0.00	0.00	0.00	0.00	0.00	0.00
NBPA 3	0.00	0.00	0.00	0.00	0.00	0.00	0.00	0.00	0.00
NBPA 4	0.00	0.00	0.00	0.00	0.00	0.00	0.00	0.00	0.00
NBPA 5	0.00	0.00	0.00	0.00	0.00	0.00	0.00	0.00	0.00
NBPA 6	0.00	0.00	0.00	0.00	0.00	0.00	0.00	0.00	0.00
NBPA 7	0.00	0.00	0.00	0.00	0.00	0.00	0.00	0.00	0.00
NBPA 8	0.00	0.00	0.00	0.00	0.00	0.00	0.00	0.00	0.00
NBPA 9	0.00	0.00	0.00	0.00	0.00	0.00	0.00	0.00	0.00
NBPA 10	0.00	0.00	0.00	0.00	0.00	0.00	0.00	0.00	0.00
NBPA 11	0.00	0.00	0.00	0.00	0.00	0.00	0.00	0.00	0.00
NBPA 12	0.00	0.00	0.00	0.00	0.00	0.00	0.00	0.00	0.00
NBPA 13	0.00	0.00	0.00	0.00	0.00	0.00	0.00	0.00	0.00
NBPA 14	0.00	0.00	0.00	0.00	0.00	0.00	0.00	0.00	0.00
NBPA 15	0.00	0.00	0.00	0.00	0.00	0.00	0.00	0.00	0.00
NBPA 16	0.00	0.00	0.00	0.00	0.00	0.00	0.00	0.00	0.00
NBPA 17	0.00	0.00	0.00	0.00	0.00	0.00	0.00	0.00	0.00
NBPA 18	0.00	0.00	0.00	0.00	0.00	0.00	0.00	0.00	0.00
NBPA 19	0.00	0.00	0.00	0.00	0.00	0.00	0.00	0.00	0.00
NBPA 20	0.00	0.00	0.00	0.00	0.00	0.00	0.00	0.00	0.00
NBPA 21	0.00	0.00	0.00	0.00	0.00	0.00	0.00	0.00	0.00
NBPA 22	0.00	0.00	0.00	0.00	0.00	0.00	0.00	0.00	0.00
NBPA 23	0.00	0.00	0.00	0.00	0.00	0.00	0.00	0.00	0.00
NBPA 24	0.00	0.00	0.00	0.00	0.00	0.00	0.00	0.00	0.00
NBPA 25	0.00	0.00	0.00	0.00	0.00	0.00	0.00	0.00	0.00
NBPA 26	0.00	0.00	0.00	0.00	0.00	0.00	0.00	0.00	0.00
NBPA 27	0.00	0.00	0.00	0.00	0.00	0.00	0.00	0.00	0.00
NBPA 28	0.00	0.00	0.00	0.00	0.00	0.00	0.00	0.00	0.00
NBPA 29	0.00	0.00	0.00	0.00	0.00	0.00	0.00	0.00	0.00
NBPA 30	0.00	0.00	0.00	0.00	0.00	0.00	0.00	0.00	0.00

Subject	Specific IgE (OD)								
	Bet v 1	F1	F2	P1	P2	P3	P6	P4	P5
NA 1	0.00	0.00	0.00	0.00	0.00	0.00	0.00	0.00	0.00
NA 2	0.00	0.00	0.00	0.00	0.00	0.00	0.00	0.00	0.00
NA 3	0.00	0.00	0.00	0.00	0.00	0.00	0.00	0.00	0.00
NA 4	0.00	0.00	0.00	0.00	0.00	0.00	0.00	0.00	0.00
NA 5	0.00	0.00	0.00	0.00	0.00	0.00	0.00	0.00	0.00
NA 6	0.00	0.00	0.00	0.00	0.00	0.00	0.00	0.00	0.00
NA 7	0.00	0.00	0.00	0.00	0.00	0.00	0.00	0.00	0.00
NA 8	0.00	0.00	0.00	0.00	0.00	0.00	0.00	0.00	0.00
NA 9	0.00	0.00	0.00	0.00	0.00	0.00	0.00	0.00	0.00
NA 10	0.00	0.00	0.00	0.00	0.00	0.00	0.00	0.00	0.00
NA 11	0.00	0.00	0.00	0.00	0.00	0.00	0.00	0.00	0.00
NA 12	0.00	0.00	0.00	0.00	0.00	0.00	0.00	0.00	0.00
NA 13	0.00	0.00	0.00	0.00	0.00	0.00	0.00	0.00	0.00
NA 14	0.00	0.00	0.00	0.00	0.00	0.00	0.00	0.00	0.00
NA 15	0.00	0.00	0.00	0.00	0.00	0.00	0.00	0.00	0.00
NA 16	0.00	0.00	0.00	0.00	0.00	0.00	0.00	0.00	0.00
NA 17	0.00	0.00	0.00	0.00	0.00	0.00	0.00	0.00	0.00
NA 18	0.00	0.00	0.00	0.00	0.00	0.00	0.00	0.00	0.00
NA 19	0.00	0.00	0.00	0.00	0.00	0.00	0.00	0.00	0.00
NA 20	0.00	0.00	0.00	0.00	0.00	0.00	0.00	0.00	0.00
NA 21	0.00	0.00	0.00	0.00	0.00	0.00	0.00	0.00	0.00
NA 22	0.00	0.00	0.00	0.00	0.00	0.00	0.00	0.00	0.00
NA 23	0.00	0.00	0.00	0.00	0.00	0.00	0.00	0.00	0.00
NA 24	0.00	0.00	0.00	0.00	0.00	0.00	0.00	0.00	0.00
NA 25	0.00	0.00	0.00	0.00	0.00	0.00	0.00	0.00	0.00
NA 26	0.00	0.00	0.00	0.00	0.00	0.00	0.00	0.00	0.00
NA 27	0.00	0.00	0.00	0.00	0.00	0.00	0.00	0.00	0.00
NA 28	0.00	0.00	0.00	0.00	0.00	0.00	0.00	0.00	0.00
NA 29	0.00	0.00	0.00	0.00	0.00	0.00	0.00	0.00	0.00
NA 30	0.00	0.00	0.00	0.00	0.00	0.00	0.00	0.00	0.00

Table S5. IgG levels specific for Bet v 1, Bet v 1 fragments and Bet v 1 peptides

Subject			Specific IgG (OD)									
	Gender	Age	Bet v 1	F1	F2	P1	P2	P3	P6	P4	P5	∑F1-F2
BPA 1	m	10	0.98	1.09	1.05	0.62	0.49	0.63	0.60	0.88	1.10	2.14
BPA 2	f	15	1.33	1.05	1.26	0.74	0.88	1.00	0.73	0.77	1.15	2.31
BPA 3	m	14	0.68	0.94	0.94	0.55	0.90	0.69	0.77	1.03	1.17	1.88
BPA 4	f	11	0.57	0.79	0.64	0.25	0.22	0.53	0.25	0.77	0.15	1.43
BPA 5	f	12	0.85	0.94	0.96	0.58	0.54	1.02	0.54	0.63	0.73	1.90
BPA 6	m	14	1.03	1.02	1.17	0.81	0.92	1.22	0.62	0.80	0.57	2.19
BPA 7	f	15	0.25	0.67	0.64	0.33	0.71	0.68	0.26	0.70	0.85	1.31
BPA 8	m	16	1.07	1.30	1.47	1.15	1.38	1.66	1.17	1.28	1.60	2.77
BPA 9	f	15	0.69	0.90	0.76	0.52	0.61	0.90	0.40	0.76	0.85	1.65
BPA 10	f	10	1.32	1.36	0.95	0.56	0.98	1.05	0.63	0.69	0.98	2.32
BPA 11	m	13	0.91	1.03	1.01	0.52	0.46	0.84	0.26	0.84	0.66	2.04
BPA 12	m	14	0.95	1.21	1.11	0.87	0.97	1.10	0.75	1.03	1.10	2.32
BPA 13	m	15	0.87	1.35	1.04	0.56	0.51	0.82	0.32	0.55	0.68	2.39
BPA 14	m	10	0.78	0.87	0.73	0.42	0.60	0.83	0.49	0.60	0.73	1.60
BPA 15	f	16	0.96	0.99	1.01	0.60	0.69	0.98	0.78	0.70	0.77	2.00
BPA 16	m	10	1.05	0.73	0.71	0.29	0.37	0.61	0.11	0.28	0.28	1.44
BPA 17	m	10	1.75	1.74	1.90	1.65	1.69	1.69	1.45	1.54	1.70	3.64
BPA 18	m	17	0.66	1.00	0.54	0.64	0.64	0.66	0.21	0.63	0.73	1.54
BPA 19	f	10	0.79	1.05	0.45	0.17	0.24	0.23	0.00	0.07	0.16	1.50
BPA 20	m	14	1.34	1.88	0.68	1.30	1.63	0.80	0.22	1.34	1.85	2.56
BPA 21	f	14	0.70	0.80	0.76	0.43	0.56	0.83	0.44	0.65	0.70	1.56
BPA 22	m	12	1.31	1.27	1.06	0.74	0.76	0.92	0.32	0.61	0.63	2.33
BPA 23	m	10	0.86	1.00	1.23	0.25	0.58	0.78	0.35	0.45	1.23	2.23
BPA 24	m	10	1.63	1.52	2.08	1.29	1.49	2.09	1.20	1.54	1.78	3.60
BPA 25	f	10	1.23	1.67	1.51	0.93	1.31	1.67	0.73	0.94	1.50	3.18
BPA 26	f	11	1.33	1.45	1.88	1.07	1.10	2.03	0.74	1.05	1.48	3.33
BPA 27	m	10	1.72	2.01	1.80	1.75	2.00	1.89	1.26	1.84	1.79	3.81
BPA 28	f	15	1.20	1.07	1.22	0.62	0.78	0.36	0.69	0.73	0.89	2.29
BPA 29	m	17	0.88	1.48	1.39	1.03	1.16	1.15	0.79	0.85	1.25	2.87
BPA 30	m	16	0.61	0.97	0.77	0.36	0.40	0.65	0.98	0.40	0.51	1.74
BPA 31	m	35	1.63	1.06	1.33	1.82	1.48	1.59	0.91	2.02	0.82	2.38
BPA 32	m	26	1.49	0.65	1.25	1.84	0.68	1.32	1.10	2.03	0.49	1.90
BPA 33	f	21	1.79	0.74	1.36	1.82	0.81	1.19	0.98	1.39	0.50	2.10
BPA 34	m	10	0.67	1.45	0.93	1.38	1.88	1.63	0.57	1.07	2.19	2.38
BPA 35	m	44	0.82	2.11	1.48	1.03	2.36	1.67	1.25	1.40	2.06	3.59
BPA 36	m	34	1.72	1.45	1.21	1.73	1.87	1.73	0.75	1.67	1.45	2.66
BPA 37	m	33	1.41	1.39	0.91	1.76	1.67	0.86	0.55	1.02	1.06	2.30
BPA 38	m	12	1.91	1.39	1.93	2.17	1.79	2.22	1.72	1.76	1.04	3.31
BPA 39	m	38	1.78	0.00	0.00	1.96	0.55	0.49	0.00	1.40	0.00	0.00

Subject			Specific IgG (OD)									
	Gender	Age	Bet v 1	F1	F2	P1	P2	P3	P6	P4	P5	∑F1-F2
NBPA 1	m	16	0.65	0.71	0.52	0.37	0.06	0.43	0.00	0.61	0.73	1.23
NBPA 2	m	15	1.25	1.29	1.72	0.96	1.18	0.81	0.94	1.05	1.42	3.00
NBPA 3	f	10	0.97	1.43	1.31	1.36	0.78	0.94	0.96	1.44	1.46	2.74
NBPA 4	f	11	0.97	1.40	1.72	0.92	1.05	1.14	0.95	0.92	1.05	3.12
NBPA 5	m	11	0.53	0.94	0.76	0.37	0.44	0.35	0.35	0.40	0.63	1.70
NBPA 6	m	14	0.65	1.01	1.21	0.88	0.86	0.57	0.54	0.86	0.57	2.21
NBPA 7	m	10	0.57	1.38	0.98	0.84	0.94	0.63	0.73	1.55	1.20	2.36
NBPA 8	f	10	0.64	1.20	1.03	0.73	0.76	0.63	0.53	0.90	0.99	2.23
NBPA 9	f	10	0.37	1.20	0.53	0.38	0.44	0.47	0.32	0.66	0.76	1.73
NBPA 10	m	15	0.64	0.93	0.89	0.50	0.57	0.47	0.34	0.57	0.85	1.81
NBPA 11	m	11	0.50	0.91	0.81	0.60	0.46	0.43	0.30	0.60	0.58	1.72
NBPA 12	m	11	1.39	1.70	1.51	1.57	1.68	1.21	1.00	1.70	1.60	3.22
NBPA 13	f	11	0.90	1.17	0.77	0.91	1.00	0.76	0.49	0.79	1.04	1.95
NBPA 14	m	11	0.38	0.80	0.67	0.57	0.65	0.52	0.40	0.66	1.03	1.47
NBPA 15	f	13	0.72	1.54	1.02	0.53	0.38	1.16	0.55	0.63	0.58	2.56
NBPA 16	f	15	0.61	0.89	0.90	0.62	0.75	0.53	0.48	0.63	0.89	1.79
NBPA 17	f	16	0.98	1.48	0.99	0.97	1.09	0.73	1.05	1.00	1.22	2.47
NBPA 18	m	16	0.48	0.68	0.35	0.51	0.51	0.43	0.12	0.32	0.37	1.04
NBPA 19	f	13	0.58	1.23	0.74	0.37	0.51	0.37	0.18	0.43	0.64	1.97
NBPA 20	f	16	0.14	0.48	0.00	0.21	0.16	0.00	0.00	0.18	0.00	0.48
NBPA 21	m	25	0.34	0.89	0.50	0.09	0.16	0.06	0.55	0.13	0.16	1.39
NBPA 22	f	14	1.05	1.59	1.09	0.52	0.54	0.35	0.09	0.52	0.38	2.68
NBPA 23	m	10	0.13	0.50	0.51	0.16	0.33	0.09	0.15	0.27	0.49	1.01
NBPA 24	f	11	0.61	1.21	1.01	0.59	0.67	0.52	0.69	0.67	0.97	2.23
NBPA 25	m	16	0.35	0.77	0.63	0.22	0.51	0.17	0.23	0.32	0.68	1.41
NBPA 26	f	10	0.78	1.02	1.36	0.71	0.91	0.51	0.45	0.69	1.23	2.38
NBPA 27	m	13	1.21	1.37	0.93	1.31	1.06	0.88	0.82	1.16	1.15	2.30
NBPA 28	m	10	0.93	1.33	1.24	0.98	1.12	0.59	0.71	0.83	1.47	2.57
NBPA 29	f	14	1.10	1.28	1.42	1.18	1.34	0.71	0.86	0.99	1.50	2.70
NBPA 30	m	30	0.71	1.20	0.68	0.27	0.34	0.24	0.73	0.39	0.45	1.89

Subject			Specific IgG (OD)									
	Gender	Age	Bet v 1	F1	F2	P1	P2	P3	P6	P4	P5	∑F1-F2
NA 1	f	13	1.00	1.20	0.94	1.08	0.64	0.72	0.66	0.93	1.39	2.87
NA 2	m	16	0.64	0.98	0.27	0.37	0.07	0.28	0.35	0.60	0.81	1.97
NA 3	m	15	1.45	1.70	1.23	1.50	1.51	1.32	1.65	1.57	1.44	3.65
NA 4	m	13	1.45	1.60	1.30	1.06	1.78	1.35	1.28	1.52	1.25	3.63
NA 5	m	11	1.07	1.29	0.98	0.84	1.41	1.09	1.11	1.25	1.71	2.99
NA 6	m	14	0.90	1.23	0.90	1.03	1.10	0.85	0.70	1.01	1.01	2.85
NA 7	m	29	1.32	1.52	0.85	1.37	1.54	1.25	1.45	1.64	1.58	3.09
NA 8	f	11	0.85	1.42	0.41	0.28	0.31	0.22	0.20	0.54	0.44	2.56
NA 9	f	14	0.74	1.45	0.75	0.88	0.85	0.75	0.72	1.20	1.21	2.92
NA 10	f	11	0.42	1.05	0.42	0.40	0.39	0.25	0.30	0.47	0.60	2.19
NA 11	f	19	0.85	1.25	0.84	0.76	0.96	0.67	0.59	0.82	0.95	2.81
NA 12	f	15	1.25	1.47	1.18	1.26	1.35	1.11	1.07	1.26	0.92	3.37
NA 13	m	14	0.70	1.20	0.58	0.55	0.78	0.37	0.51	0.69	0.95	2.50
NA 14	m	16	0.27	1.07	0.34	0.17	0.30	0.28	0.05	0.23	0.18	2.14
NA 15	f	15	0.31	0.88	0.36	0.14	0.07	0.20	0.16	0.34	0.24	1.96
NA 16	f	29	0.21	0.74	0.15	0.03	0.12	0.22	0.00	0.11	0.09	1.61
NA 17	f	45	1.31	1.83	1.26	1.36	1.57	1.28	1.39	1.38	1.36	3.81
NA 18	f	41	0.81	0.88	0.65	0.81	0.81	0.60	0.53	0.71	0.77	2.24
NA 19	f	37	0.75	1.41	0.63	0.49	0.52	0.27	0.13	0.59	0.52	2.76
NA 20	f	44	0.88	0.46	0.64	1.11	0.63	0.45	0.00	0.95	0.17	1.81
NA 21	f	16	1.03	1.10	0.45	0.33	0.50	0.42	0.40	0.59	0.56	2.27
NA 22	f	17	1.48	1.94	0.81	0.43	0.44	0.29	1.16	0.37	0.32	3.47
NA 23	f	18	0.64	0.89	0.40	0.60	0.92	0.43	0.50	0.67	1.19	2.02
NA 24	f	14	0.53	1.03	0.50	0.46	0.64	0.47	0.66	0.77	0.84	2.26
NA 25	f	25	0.31	0.97	0.11	0.15	0.21	0.02	0.21	0.37	0.30	1.80
NA 26	m	25	0.58	1.22	0.51	0.59	1.04	0.42	0.39	0.65	1.21	2.44
NA 27	m	23	1.80	1.56	1.33	1.68	1.57	1.41	1.32	1.57	1.57	3.61
NA 28	f	34	0.79	1.03	0.47	0.53	0.61	0.37	0.49	0.60	0.58	2.22
NA 29	f	27	0.48	0.77	0.40	0.40	0.71	0.21	0.52	0.45	0.61	1.89
NA 30	m	27	0.67	0.91	0.35	0.36	0.65	0.36	0.63	0.39	0.64	1.98

Table S6. IgG_1 and IgG_4 levels specific for Bet v 1 and Bet v 1 fragments

Subject	Gender	Age	Specific IgG_1 (OD)				Specific IgG_4 (OD)			
			Bet v 1	F1	F2	∑F1-F2	Bet v 1	F1	F2	∑F1-F2
BPA 1	m	10	0.12	0.00	0.00	0.00	2.23	0.50	0.16	0.67
BPA 2	f	15	0.04	0.00	0.00	0.00	0.99	0.00	0.07	0.07
BPA 3	m	14	0.00	0.00	0.00	0.00	0.35	0.00	0.00	0.00
BPA 4	f	11	0.44	0.00	0.00	0.00	1.90	0.00	0.00	0.00
BPA 5	f	12	0.00	0.00	0.00	0.00	0.46	0.00	0.00	0.00
BPA 6	m	14	0.00	0.00	0.00	0.00	1.72	0.19	0.31	0.50
BPA 7	f	15	0.00	0.00	0.00	0.00	0.03	0.10	0.09	0.19
BPA 8	m	16	0.31	0.00	1.59	1.59	2.27	0.00	4.01	4.01
BPA 9	f	15	0.00	0.00	1.09	1.09	0.31	0.37	4.02	4.38
BPA 10	f	10	0.14	0.00	0.00	0.00	0.00	0.00	0.46	0.46
BPA 11	m	13	0.00	0.00	1.05	1.05	1.73	1.36	3.62	4.99
BPA 12	m	14	0.00	0.00	0.11	0.11	0.80	0.00	2.22	2.22
BPA 13	m	15	0.00	0.08	0.00	0.08	0.09	0.00	1.19	1.19
BPA 14	m	10	0.00	0.00	0.18	0.18	1.55	0.00	0.17	0.17
BPA 15	f	16	0.00	0.00	0.00	0.00	0.61	0.00	2.39	2.39
BPA 16	m	10	0.23	0.00	0.03	0.03	0.95	0.00	2.34	2.34
BPA 17	m	10	0.00	0.00	0.10	0.10	0.96	0.00	2.17	2.17
BPA 18	m	17	0.00	0.00	0.00	0.00	0.00	0.00	0.00	0.00
BPA 19	f	10	0.00	0.00	0.00	0.00	0.53	0.00	0.34	0.34
BPA 20	m	14	0.00	0.00	0.13	0.13	0.81	0.14	3.13	3.27
BPA 21	f	14	0.00	0.00	0.16	0.16	0.15	0.00	0.90	0.90
BPA 22	m	12	0.09	0.00	0.21	0.21	1.08	0.29	1.95	2.24
BPA 23	m	10	0.33	0.01	0.00	0.01	2.25	1.73	1.70	3.43
BPA 24	m	10	0.30	0.08	0.00	0.08	2.54	2.93	2.36	5.29
BPA 25	f	10	0.00	0.00	0.00	0.00	0.00	0.00	0.72	0.72
BPA 26	f	11	0.00	0.00	0.00	0.00	0.00	0.00	1.91	1.91
BPA 27	m	10	0.00	0.00	0.44	0.44	0.00	0.00	0.65	0.65
BPA 28	f	15	0.00	0.00	0.00	0.00	0.41	0.00	0.00	0.00

Subject	Gender	Age	Specific IgG_1 (OD)				Specific IgG_4 (OD)			
			Bet v 1	F1	F2	∑F1-F2	Bet v 1	F1	F2	∑F1-F2
NA 1	f	13	0.36	0.36	0.62	0.97	0.43	0.35	0.35	0.70
NA 2	m	16	0.23	0.27	0.74	1.01	0.10	0.12	0.12	0.23
NA 3	m	15	0.27	0.67	0.31	0.97	0.14	0.14	0.12	0.26
NA 4	m	13	0.26	0.26	0.23	0.50	0.27	0.28	0.29	0.56
NA 5	m	11	0.36	0.42	0.57	0.99	0.17	0.19	0.13	0.32
NA 6	m	14	0.45	0.37	0.30	0.67	0.12	0.14	0.11	0.25
NA 7	m	29	0.25	0.29	0.27	0.56	0.10	0.11	0.10	0.20
NA 8	f	11	0.37	0.40	0.32	0.72	0.45	0.41	0.28	0.70
NA 9	f	14	0.26	0.27	0.21	0.48	0.37	0.34	0.21	0.55
NA 10	f	11	0.54	1.11	1.26	2.37	0.55	0.72	0.50	1.22
NA 11	f	19	0.42	1.00	0.59	1.58	0.18	0.21	0.22	0.44
NA 12	f	15	0.22	1.53	0.26	1.80	0.62	0.70	0.53	1.23
NA 13	m	14	0.17	0.38	0.20	0.58	0.62	0.27	0.32	0.59
NA 14	m	16	0.21	0.30	0.22	0.51	0.14	0.14	0.15	0.30
NA 15	f	15	0.19	0.23	0.16	0.39	0.12	0.13	0.12	0.25
NA 16	f	29	0.17	0.28	0.17	0.45	0.28	0.28	0.21	0.50
NA 17	f	45	0.17	0.32	0.17	0.49	0.11	0.11	0.12	0.23
NA 18	f	41	0.24	0.40	0.27	0.67	0.24	0.34	0.21	0.55
NA 19	f	37	0.14	0.18	0.23	0.41	1.44	1.83	2.69	4.53
NA 20	f	44	0.11	0.14	0.17	0.31	1.27	1.27	1.91	3.18
NA 21	f	16	0.11	0.16	0.12	0.28	1.39	1.39	1.48	2.86
NA 22	f	17	0.14	0.21	0.15	0.36	0.73	0.79	1.12	1.91
NA 23	f	18	0.28	0.40	0.14	0.54	0.75	0.73	0.81	1.55
NA 24	f	14	0.22	0.26	0.16	0.42	0.61	0.60	0.65	1.25
NA 25	f	25	0.13	0.39	0.17	0.56	2.09	2.40	3.95	6.35
NA 26	m	25	0.21	0.29	0.20	0.49	1.56	1.19	1.20	2.39
NA 27	m	23	0.20	0.37	0.16	0.53	2.48	4.81	5.14	9.95
NA 28	f	34	0.24	0.23	0.24	0.47	0.19	0.19	0.23	0.42
NA 29	f	27	0.18	0.27	0.25	0.52	0.24	0.31	0.37	0.68

Table S7. Inhibition of BPA patients' IgE binding to rBet v 1 by sera of NA individuals

NA serum	Bet v 1-specific IgG (OD)	BPA sera	Bet v 1-specific IgE (OD) (BPA patients)	% Inhibition	Range of inhibition (%)	Median
2	1.00	2	1.91	0	0 - 29	12
		25	0.40	28		
		27	0.65	2		
		40	NA	12		
		19	0.94	29		
3	1.81	3	1.35	22	-10 - 22	-1
		25	0.40	3		
		27	0.65	-10		
		40	NA	-6		
4	1.81	4	2.95	6	2 - 28	6
		25	0.40	2		
		19	0.94	28		
		27	0.65	21		
		41	NA	4		
8	1.21	8	3.16	4	-1 - 30	4
		19	0.94	20		
		27	0.65	30		
		40	NA	5		
		41	NA	-1		
		25	0.40	1		
15	0.67	16	2.34	14	14 - 27	17
		45	NA	20		
		44	NA	14		
		18	0.50	27		
16	0.57	17	0.66	52	-10 - 52	34
		46	NA	27		
		63	NA	2		
		47	NA	41		
		54	NA	35		
		60	NA	34		
		61	NA	-10		
17	1.67	18	0.50	44	-5 - 44	30
		45	NA	20		
		65	NA	-5		
		47	NA	40		
18	1.17	19	0.94	47	6 - 47	17
		45	NA	34		
		44	NA	22		
		18	0.50	6		
		49	NA	14		
		48	NA	6		
		51	NA	17		
		50	NA	15		
		52	NA	17		
19	1.11	20	0.82	5	-10 - 39	29
		46	NA	24		
		63	NA	-4		
		47	NA	39		
		54	NA	35		
		60	NA	38		
		61	NA	-10		
		55	NA	31		
		53	NA	27		
		48	NA	35		
21	1.39	22	1.23	31	-1 - 39	31
		46	NA	25		
		63	NA	-1		
		47	NA	37		
		54	NA	39		
		60	NA	37		
		61	NA	3		
		55	NA	26		
		48	NA	31		
24	0.89	25	0.40	45	-15 - 45	17
		45	NA	31		
		44	NA	7		
		18	0.50	13		
		56	NA	20		
		57	NA	15		
		58	NA	25		
		47	NA	-15		
		59	NA	17		
26	0.94	27	0.65	50	1 - 50	10
		45	NA	36		
		44	NA	10		
		18	0.50	1		
		49	NA	7		
		51	NA	5		
		50	NA	14		
27	2.16	28	2.22	44	6 - 44	26
		50	NA	11		
		45	NA	35		
		44	NA	6		
		18	0.50	10		
		56	NA	30		
		57	NA	22		
		58	NA	35		
